# Technology-Related Trace Elements in Paediatric Scalp-Hair Biomonitoring: Biomarker Validity, Environmental Relevance, and Implications for Risk Assessment

**DOI:** 10.3390/toxics14090813

**Published:** 2026-09-12

**Authors:** Antonio Peña-Fernández, Rafael Moreno-Gómez-Toledano, Borja Martínez-Alonso, M. Ángeles Peña Fernández

**Affiliations:** 1 Area of Legal and Forensic Medicine, Department of Surgery, Medical and Social Sciences, Faculty of Medicine and Health Sciences, University of Alcalá, Ctra. Madrid-Barcelona, Km. 33.6, 28871 Alcalá de Henares, Madrid, Spain; 2 Area of Human Anatomy and Embryology, Faculty of Medicine and Health Sciences, University of Alcalá, Ctra. Madrid-Barcelona, Km. 33.6, 28871 Alcalá de Henares, Madrid, Spain; 3 Department of Biomedical Sciences, Faculty of Pharmacy, University of Alcalá, Crta. Madrid-Barcelona Km, 33.6, 28871 Alcalá de Henares, Madrid, Spain

**Keywords:** scalp hair, children, adolescents, human biomonitoring, technology-related trace elements, rare earth elements, platinum-group elements, biomarker validity, population reference values, risk assessment

## Abstract

Technology-related trace elements (TTEs), including rare earth elements (REEs), platinum-group elements (PGEs), silver, antimony, bismuth, barium, strontium, vanadium and radionuclide-associated elements, are increasingly relevant to environmental exposure assessment. Children and adolescents are important sentinel populations, and scalp hair offers a non-invasive matrix for multielement analysis. However, a reported hair concentration may combine follicular incorporation, sweat and sebum deposition and external particles, and may also be influenced by collection, washing, cosmetic treatment and analytical contamination. Separately, left-censoring and the statistical treatment of non-detects can materially affect population summaries and comparisons. This critical narrative review integrates paediatric, occupational, environmental and multimatrix evidence through a structured evidence matrix and an element-by-element audit of biomonitoring assessment values. Hair can identify spatial contrasts and source-related patterns, but current evidence generally does not support direct inference of absorbed dose or clinical risk. In long-term occupational REE exposure, urine shows stronger exposure-response performance than blood; paired hair–urine data for thorium discriminate exposed groups without establishing quantitative equivalence; antimony findings vary by exposure setting; and barium biomonitoring equivalents are available for urine and plasma, but not hair. No health-based guidance values validated specifically for paediatric scalp hair were identified for any of the target TTEs. A seven-stage weight-of-evidence framework is proposed, positioning hair as a complementary screening and hypothesis-generating matrix within multimatrix assessment.

## 1. Introduction

The expanding use of specialised elements in digital technologies, renewable-energy systems, low-carbon transport, advanced materials and medical applications is changing the profile of environmental metal and metalloid exposure. Rare earth elements (REEs), such as lanthanum and cerium, platinum-group elements (PGEs), silver (Ag), antimony (Sb), bismuth (Bi), vanadium (V), gallium (Ga), germanium (Ge), niobium (Nb), tantalum (Ta), tungsten (W) and related elements are used in magnets, semiconductors, batteries, catalysts, photovoltaics, electronics and high-performance alloys. Their strategic importance is reflected in current critical raw materials policy, including Regulation (EU) 2024/1252 [1]. In this review, these substances are considered collectively as technology-related trace elements (TTEs), an operational exposure category that does not imply shared toxicokinetics, common toxicity or exclusively anthropogenic origin. The term TTE is therefore used here as an operational umbrella category rather than as a formal regulatory or toxicological classification. It overlaps with concepts such as critical raw materials, technology-critical elements, emerging contaminants, potentially toxic elements and essential trace elements, but technological relevance does not imply toxicity, and a toxic element need not be technologically critical. Environmental-health interpretation must remain element- and species-specific and consider concentration, chemical form, particle size, bioavailability, exposure route and duration and developmental susceptibility.

Human exposure may arise across the full technological life cycle, from extraction, beneficiation and refining to manufacturing, product use, mechanical wear, recycling and disposal. These releases coexist with geogenic inputs from rocks, soils, water and mineral dust. Consequently, the detection of a TTE in an environmental or biological sample is not, by itself, evidence of technological contamination. Source interpretation requires information on local geology, environmental media, particle characteristics, product use, exposure pathways and the spatial and temporal correspondence between the suspected source and the biological sample.

Children and adolescents are relevant to this emerging exposure landscape for two distinct reasons. First, their exposure patterns may differ from those of adults because of greater contact with floors, settled dust, soil and outdoor surfaces, age-specific hand-to-mouth activity, diet and, for some exposure media, higher intake relative to body weight. Second, developmental stage may modify susceptibility to adverse effects. These considerations are related but distinct and do not imply that paediatric exposure is universally higher for every TTE. Mobility, school and transport environments, cosmetic practices and consumer-product use also change across childhood and adolescence [2]. Paediatric biomonitoring must therefore distinguish developmental groups and account for the environments in which young people actually spend time.

Human biomonitoring can integrate exposure from several sources and routes, but its interpretative value depends on the suitability of the measured biomarker for the element, chemical species, exposure route, biological half-life and decision question [3,4]. Blood and urine remain the most established matrices for many inorganic contaminants. Scalp hair is nevertheless attractive in paediatric research because collection is non-invasive, generally well accepted, compatible with repeated or school-based sampling and suitable for storage and retrospective multielement analysis [5,6].

These practical advantages have sometimes encouraged a stronger interpretation than the evidence supports. A concentration measured in scalp hair may include elements incorporated during follicular growth, material deposited through sweat or sebum and particles or products retained on or within the hair shaft. Washing does not necessarily remove all external material, while cosmetic treatment, pigmentation, collection tools, digestion vessels, procedural blanks and analytical detection capability can materially affect the reported result [6,7,8]. Hair is therefore not a single, universally defined biomarker: depending on the element and study design, it may function as an internal-exposure indicator, an environmental-contact matrix, a passive particle collector or a composite of these processes.

The distinction between analytical detectability and biomarker validity is particularly important for TTEs, many of which occur at ultra-trace concentrations and lack matrix-matched reference materials, established toxicokinetic models or paediatric health-based values. Direct comparisons show that hair does not consistently correspond with internal matrices. In children, hair concentrations have shown important limitations as biomarkers for several toxic and essential elements [9]. In adults, hair Pb concentrations were only weakly related to blood Pb concentrations, while hair Cu, Mn and Sr concentrations were not significantly related to the corresponding blood concentrations. Moreover, geographical differences observed in hair were not consistently mirrored in blood, indicating that an area-related pattern observed in one matrix should not automatically be interpreted as the same systemic exposure pattern [10,11]. Thus, an analytically valid hair concentration may still be unsuitable for estimating absorbed dose or health risk.

The literature is currently fragmented across environmental-exposure studies, analytical-method papers, studies reporting population reference distributions or intervals and biomonitoring-guidance frameworks. Population reference distributions describe concentration variability within a defined population under a specified analytical workflow and may indicate whether an observation is common or unusual in that context; they are not health-based toxicity thresholds. A focused synthesis is needed to determine which conclusions are supported for paediatric TTE hair data, which require corroboration in blood, urine or environmental media, and which remain scientifically unjustified. The primary aim of this critical narrative review is to evaluate the validity and appropriate interpretative role of paediatric scalp hair for technology-related trace elements. Specifically, the review addresses four linked questions: (i) what environmental and biological processes determine the concentration reported in hair; (ii) which TTEs and exposure settings currently support occurrence, spatial or source-pattern interpretation, and which provide evidence of internal exposure; (iii) how analytical quality, left-censoring, population reference distributions and matrix-specific biomonitoring values constrain risk interpretation; and (iv) what evidence is required to progress from analytical detection to source attribution, toxicological relevance and proportionate public-health action. On this basis, the review develops a structured evidence map, audits available biomonitoring assessment values and proposes a seven-stage weight-of-evidence framework for research and practice.

## 2. Materials and Methods

This structured critical narrative review used an author-curated full-text evidence corpus that was deduplicated and supplemented through targeted searches of PubMed, Google Scholar and publisher databases, together with backward and forward citation tracking; searches were updated to 6 August 2026. Search concepts combined scalp or human hair; children, adolescents and paediatric populations; human biomonitoring and exposure biomarkers; technology-related or technology-critical elements and the individual target elements (REEs, PGEs, Ag, Sb, Bi, Ba, Sr, V, U, Th, Ga, Ge, Nb, Ta and W); and terms addressing external contamination, washing, ICP-MS or ICP-MS/MS, analytical quality assurance, reference distributions, biomonitoring guidance values, toxicokinetics, health-risk assessment and multimatrix comparisons. Priority was given to peer-reviewed paediatric studies, environmentally or occupationally exposed populations, multimatrix investigations, analytical-validity studies, paediatric reference-distribution studies and authoritative or peer-reviewed biomonitoring frameworks; adult and occupational evidence was retained where paediatric evidence was unavailable or where it provided important toxicokinetic, analytical or exposure–response information. The final structured evidence matrix comprised 61 unique core sources: 32 human or exposure-setting studies (Table S1A), 20 multimatrix, analytical or statistical-validity sources (Table S1B) and 9 biomonitoring or risk-interpretation frameworks (Table S1C). Contextual, policy and assessment-value sources were used separately where relevant and were not assigned the same role as core biomarker-validity evidence. Because literature identification was iterative and targeted rather than a prospectively logged systematic-screening workflow, candidate records and exclusion decisions were not recorded in a form that permits a defensible retrospective count of all records retrieved and excluded; accordingly, we report the exact composition of the retained core corpus rather than reconstructing a PRISMA-style flow retrospectively. Studies were critically appraised according to design, population, sample size, age and demographic structure, the definition and magnitude of differences between exposure groups or levels, comparison populations, spatial and temporal correspondence, individual or pooled sampling, collection and washing protocols, cosmetic treatment, analytical validation, procedural-blank control, matrix-matched reference materials, LOD/LOQ definitions, treatment of censored observations, complementary matrices, dietary and behavioural factors that could influence exposure or confound biomarker associations, source attribution and the distinction between statistical association and toxicological interpretation. Greater evidential weight was assigned to studies combining independent biological or environmental matrices, clearly characterised differences in exposure, appropriate QA/QC and biologically plausible exposure–response relationships. A separate element-by-element audit assessed health-based biomonitoring values, biomonitoring equivalents, occupational biological assessment values and values validated specifically for paediatric scalp hair; assessment values were retained only for the biological matrix and interpretative purpose for which they had been derived. Statistical or background reference values, air occupational exposure limits and commercial laboratory interpretive ranges not derived as authoritative health-based biomonitoring thresholds were not classified as health-based hair values (Tables S2 and S3). Quantitative meta-analysis and numerical risk-of-bias scoring were not applied because of substantial methodological heterogeneity. During preparation of the review, ChatGPT (OpenAI; GPT-5.6 and the integrated OpenAI image-generation capability) was used to support evidence organisation, language drafting, structural refinement and graphical development. Scientific concepts, evidence selection, interpretation and intended figure content were defined and verified by the authors; no generative-AI tool was used to generate or fabricate experimental data, numerical results or statistical analyses or to determine study eligibility autonomously, and all AI-assisted outputs were critically reviewed by the authors.

## 3. Environmental Relevance of Technology-Related Trace Elements

Table 1 summarises the principal uses, environmental sources, defensible interpretative roles of scalp-hair measurements and major limitations for the technology-related element groups considered in this review.

**Figure 1:**
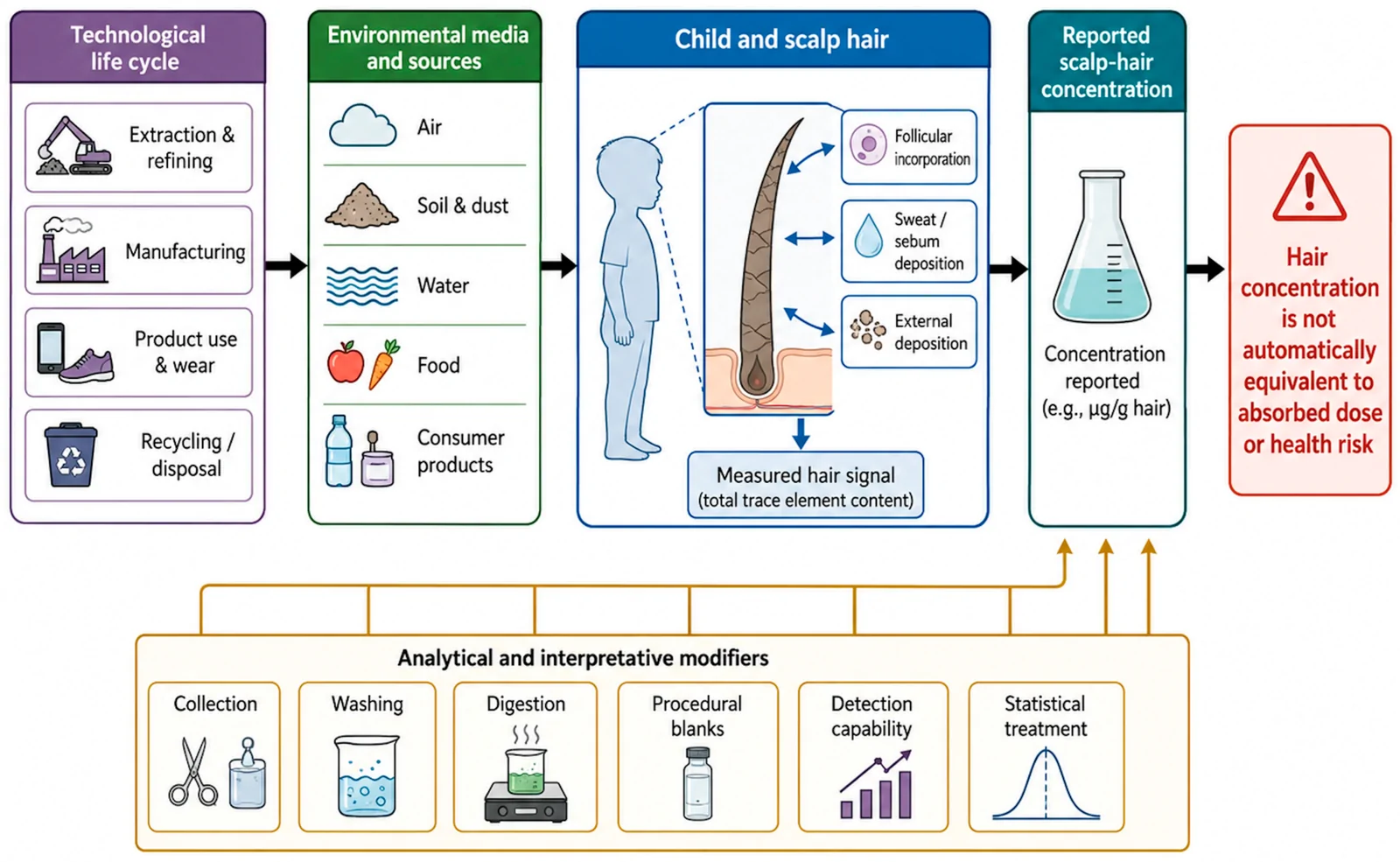
Conceptual pathway linking the technological life cycle of trace elements with paediatric scalp-hair measurements. Elements released through extraction, refining, manufacturing, product use, wear, recycling and disposal may reach children through air, soil, dust, water, food and consumer products. The concentration measured in hair may include contributions from follicular incorporation, sweat, sebum and external deposition. Collection, washing, digestion, analytical blanks, detection capability and statistical treatment further influence the reported result. Consequently, a hair concentration is not automatically equivalent to absorbed dose or health risk. The principal exposure contexts summarised in Table 1—mining and mineral processing, urban wear and combustion, consumer-product contact and recycling or electronic-waste handling—are encompassed within this life-cycle framework.

Across the evidence summarised in Table 1 and Figure 1, several recurring exposure contexts emerge. Reviews of human REE exposure and toxicity provide the broader context for interpreting hair findings [12,13]. Under pronounced exposure differences associated with REE mining or smelting, scalp hair can preserve spatial and geochemical signals, but available multimatrix data do not establish proportionality to systemic uptake; in long-term occupational REE exposure, urine currently shows stronger exposure–response performance than blood [14,15,16,17,18]. Additional hair-based REE studies in children, adolescents and maternal populations broaden the evidence base but do not establish quantitative hair–dose validity [19,20,21]. For PGEs, environmental-fate and human-hair evidence supports occurrence and source-pattern interpretation but not dose inference [22,23,24]. In urban, industrial and consumer-product settings, Ag, Sb, Bi, Ba, Sr and V can show geographical or source-related patterns, but external deposition, cosmetic or product contact, geogenic and dietary inputs, analytical censoring and matrix-specific kinetics constrain dose interpretation [25,26,27,28,29,30,31,32,33,34]. At the ultra-trace concentrations relevant to PGEs, instrumental carry-over or memory can additionally affect results when analyte retained from preceding measurements is not removed adequately during washout. For U and Th, total hair concentrations do not determine isotope composition, chemical species or radiological dose; paired hair–urine data for Th can distinguish exposure groups without demonstrating quantitative equivalence [35,36]. Evidence for Ga, Ge, Nb, Ta and W remains sparse, whereas electronic-waste exposure represents a complex mixture that requires environmental characterisation and validated complementary matrices rather than hair alone [37]. Across these settings, scalp hair is therefore most defensible as a screening and source-pattern matrix rather than as a stand-alone surrogate of absorbed dose or clinical risk.

## 4. Scalp Hair as a Paediatric Biomonitoring Matrix

### 4.1. Practical Advantages

Hair sampling is painless, non-invasive and generally acceptable in school, community and longitudinal research. It does not require venepuncture, immediate centrifugation or urine-collection facilities. Samples require little storage space and may be transported under less demanding conditions than many fluid matrices.

Advances in minimally invasive biospecimen collection are particularly relevant to children’s exposome research [38]. Hair can complement dried capillary blood, saliva, deciduous teeth, urine and other matrices by providing a different exposure window and practical collection profile.

A small mass of hair can support multielement analysis using ICP-MS or ICP-MS/MS. Archived samples can also be reanalysed as analytical technology improves or new environmental questions emerge.

### 4.2. Biological Incorporation

Hair is produced within follicles through cellular proliferation and keratinisation. Elements circulating in blood may enter the growing shaft and interact with keratin, sulfhydryl groups, melanin, structural proteins and intracellular constituents.

This pathway provides biological plausibility for hair as an exposure matrix. It does not establish a constant or linear relationship between hair concentration and circulating concentration. Elemental incorporation depends on chemical form, protein and melanin binding, circulating concentration, hair-growth phase, follicular physiology, nutritional status, age, sex and individual hair characteristics. Validation for one element cannot be transferred automatically to another.

### 4.3. Sweat and Sebum

After emerging from the scalp, hair is exposed to sweat and sebum. Elements excreted through these pathways may deposit on or diffuse into the cuticle.

This contribution may indirectly reflect internal exposure, but its magnitude is influenced by perspiration, scalp physiology, hygiene and hair products. It is not equivalent to follicular incorporation.

### 4.4. External Deposition

External deposition is a major determinant of hair-associated elements. Particles from soil, road dust, mining emissions, industrial aerosols, smoke, water and cosmetics may adhere to the cuticle or become embedded within damaged structures.

External material may still be environmentally informative. Hair-associated dust may indicate that a child occupied a contaminated environment and could have inhaled or ingested particles from the same source. However, the bulk hair concentration cannot then be assumed to represent systemic absorption.

The endogenous–exogenous distinction is therefore not binary. Interpretation should consider the operationally measured fraction, whether the research question concerns internal dose or environmental contact, whether surface particles or wash fractions were characterised, whether the elemental pattern resembles local soil or dust, whether blood or urine supports systemic uptake and whether the finding persists in a newly collected sample.

### 4.5. Exposure Window and Segmental Interpretation

Hair is often described as a long-term or cumulative matrix. More precisely, a hair segment integrates processes affecting that segment during and after formation.

Approximate scalp-hair growth rates can support broad exposure windows, but growth varies among individuals, anatomical regions and growth phases. Segmental analysis should not be assigned exact calendar dates without individual growth information. Distal segments also have a longer opportunity for external deposition than proximal segments.

### 4.6. Hair Colour, Melanin and Fibre Morphology

Some elements have an affinity for melanin, so pigmentation may influence incorporation independently of environmental exposure. Hair-fibre morphology also deserves consideration. Fibre geometry, curvature, cross-sectional characteristics, mechanical behaviour and water-swelling properties vary among individuals and have been shown to differ in their distributions among hair phenotypes from populations of different ancestry [39,40]. Such physical characteristics could influence surface interactions, retention of externally deposited material and responses to washing or cosmetic treatment, although direct evidence quantifying their effect on trace-element biomonitoring remains limited. Broad ethnic categories should therefore not be used as proxies for elemental uptake. Where relevant, studies should instead record directly observable hair characteristics, including natural colour, curvature or texture, treatment history and visible damage, together with appropriate demographic information. Across the evidence reviewed here, detailed hair morphology and ancestry or ethnicity were not reported or incorporated consistently into washing validation or multivariable interpretation. This represents a limitation of the current evidence base and should be addressed in future paediatric biomonitoring studies.

### 4.7. Cosmetic Treatment

Dyeing, bleaching, straightening and permanent treatments alter cuticle integrity, porosity and chemical composition. They can introduce elements directly or modify susceptibility to environmental deposition and washing.

Treated hair should be excluded where appropriate, analysed separately, included as a covariate or examined in sensitivity analyses. Cosmetic treatment is especially important for Ag and Bi and may also alter results for elements not directly present in the product by changing the structure of the hair shaft.

### 4.8. Paediatric Ethics and Communication

Although hair sampling presents minimal physical risk, paediatric studies require ethical oversight, parental or guardian consent and age-appropriate assent.

Cultural and personal sensitivities concerning hair should be respected. Sampling should be discreet and avoid visible cosmetic alteration.

The return of individual results requires particular care. Families may interpret an elevated numerical result as a diagnosis even where no health-based value exists. Consent documentation should clarify whether individual results will be returned, which comparison distribution will be used, whether the result has recognised clinical meaning, when repeat analysis or recollection will occur and what environmental or clinical follow-up is available.

### 4.9. Archived Hair

Archived hair can provide unique historical evidence. Its use requires documentation of the original collection date, anatomical location, segment length, storage container, storage environment, previous handling, sample mass, chain of custody, ethical authority for secondary analysis and comparability between historical and contemporary methods.

Harmonisation of storage, shipment and associated data management is a wider priority for human biomonitoring and biobanking [41]. Metals are not expected to undergo biological degradation in stored keratin, but contamination, repeated handling and undocumented preparation remain possible. Archived hair should therefore be described as a retrospective occurrence baseline rather than an exact reconstruction of absorbed historical dose.

## 5. Pre-Analytical and Analytical Validity

### 5.1. Biomarker Fitness

Before hair is collected, investigators should define the decision question. Hair may be suitable for occurrence characterisation, demographic comparison, geographical screening, source investigation, intervention follow-up, identification of unusual observations and selection for complementary biomonitoring. Suitability for estimation of internal dose or clinical interpretation requires substantially stronger element-specific evidence.

### 5.2. Sampling Location and Segment

Samples should usually be collected close to the scalp from a consistent region, commonly the posterior vertex. The proximal end should be identified. The anatomical location, segment length, sample mass, proximal–distal orientation, collection date, collection device, storage container and storage conditions should be reported.

### 5.3. Collection-Tool Contamination

Collection tools can contaminate ultra-trace analyses. Runkel et al. demonstrated that stainless-steel scissors may contribute materially to some elemental signals [42].

Tool validation should include unused-tool blanks, repeated cutting of clean materials, assessment of tool-alloy composition, cleaning-efficiency experiments, field-transport controls and evaluation of ceramic or alternative materials. A tool suitable for conventional metals may not be suitable for Nb, W, Co, Cr or other technology-related elements.

### 5.4. Participant Metadata

Minimum participant-level information should include age, sex, natural hair colour, cosmetic treatment, washing frequency, swimming, water contact, hair and scalp products, medication, supplements, diet, seafood consumption, drinking-water source, residence, residential history, school location, activity spaces, proximity to traffic or industry, parental occupations and relevant hobbies or consumer-product use.

Failure to collect these variables limits source attribution and may leave residual confounding that cannot be corrected statistically.

### 5.5. Washing Procedures

No washing method has been shown to remove all external contamination while preserving all internally associated elements.

Morton et al. demonstrated that different procedures remove externally bound elements with varying efficiency [43]. Verrey et al. proposed a sequential washing approach for a broad inorganic panel [44]. David et al., using LA-ICP-MS, showed that pre-cleaning did not improve every application and could alter spatial information [45].

These findings are not necessarily contradictory because bulk digestion and spatially resolved analysis measure different operational quantities. A washing protocol should report reagent identity and purity, sequence, number of cycles, reagent volume, duration, agitation, rinsing, drying, vessel material and whether wash solutions were analysed. Washed hair should not be described as a purely endogenous matrix unless this has been demonstrated experimentally.

### 5.6. Digestion and Instrumentation

Closed-vessel microwave digestion with high-purity acids is commonly used for complete matrix destruction. Methods should report sample mass, acid composition and grade, vessel material, digestion temperature and pressure, final dilution, isotopes, collision or reaction gases, correction equations, internal standards, calibration range, drift correction, carry-over, washout time and result-acceptance criteria.

Ultra-trace REEs, PGEs and technology-related elements are especially susceptible to contamination, instrumental carry-over/memory, incomplete washout and polyatomic interferences. Method performance at conventional-metal concentrations does not establish performance at the much lower concentrations of many emerging elements.

Conventional complete acid digestion followed by ICP-MS or ICP-MS/MS provides an operational total-element concentration and does not preserve the original chemical species. This distinction is important for elements such as Sb, V and U because oxidation state and chemical speciation can influence environmental behaviour, bioavailability, toxicokinetics and toxicological interpretation [6,27,46]. When the decision question concerns internal dose or toxicological relevance rather than occurrence or source screening, species-resolved analysis or complementary speciation in an appropriately validated internal matrix should therefore be considered. Speciation requires dedicated sample preservation and separation procedures and cannot be reconstructed retrospectively from a complete acid digest.

### 5.7. Procedural Blanks

Full procedural blanks should accompany each relevant preparation batch. Reagent blanks alone do not capture contamination introduced by cutting, washing, vessels, handling, digestion, dilution and the laboratory environment.

Blank assessment should examine central tendency, dispersion, temporal drift, contaminated vessels, reagent changes, preparation batches and anomalous blank populations. A single global blank correction may be inappropriate where batches differ materially.

### 5.8. Negative Blank-Corrected Values

Negative blank-corrected concentrations can occur when the sample signal is lower than the estimated blank contribution. These values should generally be retained in the internal analytical dataset.

Replacing negative corrected values with zero alters the distribution and may bias summary statistics, correlations and censored-data models. For presentation, values may be classified as non-detected or below quantification, provided that the original corrected data and decision rules remain auditable.

### 5.9. Certified Reference Materials and Proficiency Testing

Certified or reference materials should be selected according to the matrix and analyte. A hair certified reference material with assigned values for Pb, Cd or Hg does not validate the accuracy of REEs, PGEs or other elements lacking assigned values.

Where appropriate assigned values are unavailable, validation should combine blank stability, spike recovery, replicate precision, calibration verification, isotope agreement, alternative reference materials, interference experiments, independent-method confirmation and interlaboratory comparison. Tadić et al. identified continuing gaps in matrix reference materials and proficiency-testing schemes across human biomonitoring [47].

### 5.10. Multimatrix Analytical Validation

Goullé et al. developed and analytically validated ICP-MS methods for whole blood, plasma, urine and hair and reported population distributions for numerous elements, including V, Ga, Ge, Rb, Sr, Pd, Ag, Sb, Ba, W, Pt, Bi and U [48]. Their workflow included method-specific linearity, precision, detection and quantification limits and participation in an interlaboratory quality-control programme.

The study demonstrates that multielement measurements can be analytically validated across several biological matrices. It does not demonstrate that the resulting concentrations are biologically interchangeable. The reference datasets comprised 100 whole-blood, 100 plasma and 100 urine measurements and 45 hair measurements, and the study did not establish individual-level hair–blood or hair–urine relationships for the technology-related elements.

The reported hair distributions are valuable analytical and population comparators, but they are not health-based thresholds. They should also not be treated as direct multimatrix validation because measurement feasibility across matrices is distinct from toxicokinetic equivalence.

Olmedo et al. likewise validated methods for Cr, Cd, Mn, Ni and Pb in blood, urine, saliva and hair [49]. Analytical comparability is a prerequisite for multimatrix research, but biological equivalence must be evaluated separately.

### 5.11. Limits of Detection and Quantification

Because this article is a review, no new LOD or LOQ was calculated by the authors. For each source study, the reported definition and calculation of LOD/LOQ were considered when available; values were not retrospectively recalculated because the necessary raw blank, calibration and recovery data were generally unavailable and analytical procedures differed among studies. Failure to report how LOD/LOQ was derived was treated as an analytical-reporting limitation. For future studies, LOD and LOQ should be derived from the complete analytical procedure, expressed in final hair units and incorporate procedural-blank variation, sample mass, dilution, recovery, instrument response and predefined decision criteria. Results should distinguish non-detected observations, detections below LOQ, reliably quantified results and analytically invalid observations.

### 5.12. Left-Censored Data

Technology-related elements frequently produce left-censored datasets. Arbitrary substitution with zero, LOD/2, LOD/√2 or LOD can bias medians, variances, correlations and group comparisons, particularly where censoring is substantial or differs among groups [50].

Depending on sample size, censoring proportion, distribution and study objective, defensible approaches may include detection-frequency reporting, Kaplan–Meier estimation, regression on order statistics, maximum-likelihood estimation, censored regression, Peto–Peto or related tests and sensitivity analyses.

No method is universally optimal. When censoring is extreme, occurrence-based reporting is more defensible than estimation of a continuous distribution. An element that is 100% censored cannot support meaningful percentile, correlation, regression or principal-component analysis.

### 5.13. Multivariate and Mixture Analysis

Technology-related exposures often occur as mixtures. Road dust may contain PGEs, Sb, Cu, Zn, Ba and other wear-related elements. Mining emissions may contain REEs, U, Th, Pb, Cd or As. Electronic waste can generate product-specific mixtures.

Multivariate approaches may include correlation analysis, hierarchical clustering, principal-component analysis, elemental ratios, enrichment profiles, receptor models and supervised classification. Elements should not be included after arbitrary substitution merely to create a complete matrix, because this can generate artificial covariance and false source components. A statistical component should not be labelled as traffic, industry or geology without independent source evidence.

Table 2 presents the minimum pre-analytical, analytical and reporting requirements for paediatric scalp-hair studies.

## 6. Human Evidence Across Paediatric Populations

### 6.1. Population Patterns Across Contrasting Environmental Settings

Population-based studies consistently demonstrate that scalp hair can reveal spatial or demographic heterogeneity, but they also show why geographical proximity should not be treated as a direct surrogate for internal exposure. Drobyshev et al. identified multielement differences among 166 schoolchildren living near a toxic-waste site and in comparison areas, illustrating the feasibility of school-based screening while also highlighting limitations arising from small geographical groups, regional background variability, absence of internal biomarkers and possible external contamination [51]. In a much larger study of 1595 schoolchildren from an oil-producing region of Kazakhstan, Batyrova et al. observed age-, sex- and area-related differences, but elemental patterns did not consistently follow proximity to oil production [52]. This result suggests that mobility, diet, geology, household conditions and local activity spaces may be more informative than distance to a nominal source.

Beltrán-Ardila et al. identified school-level heterogeneity in pooled hair samples from children attending 14 schools in Bogotá [53]. Pooling provided an efficient means of geographical surveillance but prevented assessment of individual distributions, determinants or extreme observations. Taken together, these studies support hair as a population-screening matrix while demonstrating that spatial contrasts cannot be interpreted automatically as individual absorbed dose. The evidential value increases when the sampling design captures relevant activity spaces, analytical preparation is harmonised and environmental or internal matrices are collected contemporaneously.

### 6.2. European Paediatric Evidence and the Alcalá de Henares Programme

The Alcalá de Henares research programme provides one of the most extensive internally coherent series of paediatric hair studies in Europe. Early investigations characterised distributions of conventional metals and metalloids in children and adolescents and examined differences related to age, sex, residential area and urban topsoil, the latter referring to topsoil sampled within the urban environment of Alcalá de Henares rather than to rural comparison soils [54,55,56,57]. These studies established several principles that remain directly relevant to emerging elements: hair distributions are usually skewed; age and sex may alter central tendency and upper tails; residential contrasts generate source hypotheses but do not prove source attribution; and environmental and hair concentrations do not necessarily correspond directly.

Subsequent reanalysis of the archived samples extended the programme to Ag, REEs, PGEs, Ba, Sr, V and other technology-related elements [25,30,36]. The later studies addressed an analytically more difficult concentration range and placed greater emphasis on detection frequency, procedural contamination, censoring and the distinction between environmental occurrence and toxicological interpretation. The archive is scientifically valuable because it permits comparison across developmental groups and element classes within a common historical population. Its limitations are equally important: the samples were collected in 2001, the environmental datasets are not always temporally matched, blood and urine were not collected, analytical inventories vary among publications and the exposure profile predates the rapid expansion of several contemporary technologies. The earlier studies generally included 117 children and 96 adolescents, whereas the later analytical programme included up to 120 children and 97 adolescents; the exact number contributing to each element must therefore be reported rather than assuming an identical population across all publications.

Independent Spanish studies broaden the comparison. Llorente Ballesteros et al. reported reference distributions in 648 children and adolescents from Madrid, while Ruiz et al. analysed 28 elements in 419 children from a Mediterranean population without a recognised major industrial source [58,59]. Torrente et al. combined childhood hair measurements with exploratory neuropsychological assessments, and Esplugas et al. provided a rare longitudinal community perspective through repeated biomonitoring near a hazardous-waste incinerator [60,61]. The Sicilian PGE study and the Kola Peninsula REE study further extend European evidence to traffic-industrial and mining settings [20,23].

Table 3 provides a structured evidence map of selected European paediatric studies rather than a pooled reference interval. Original population characteristics, age groups, sampling periods, analytical features, units, summary statistics and detection or censoring information were retained to minimise inappropriate direct comparison among methodologically heterogeneous datasets. Arithmetic means, geometric means, medians, percentiles and censoring-aware estimates were not treated as interchangeable, and converted values, where used, were reported alongside rather than in place of the original units. Accordingly, between-study differences in reported concentrations should not be attributed to exposure alone without considering hair washing, digestion, cosmetic exclusions, sample mass, blank correction, analytical sensitivity and the treatment of non-detects. The value of this comparison lies in identifying broadly compatible concentration ranges, methodological sources of variation and technology-related elements for which independent European paediatric evidence remains unavailable.

The value of the European comparison is therefore not the derivation of a single “normal range”, but the identification of reproducible concentration ranges, methodological sources of variation and elements for which independent paediatric evidence is absent.

### 6.3. Nutritional, Behavioural and Health-Outcome Context

Elemental distributions in hair do not depend exclusively on environmental source intensity. The Malagasy REE study found generally weak relationships with age and nutritional status, suggesting that hair growth, mineral metabolism, dietary insufficiency, socioeconomic circumstances and soil or dust contact may influence the observed concentration [19]. Urinary biomonitoring in Spanish children likewise demonstrated associations between dietary groups and several metals [34]. Diet, nutritional status, drinking water and product use should consequently be treated as core exposure determinants rather than optional covariates, regardless of the biological matrix selected.

Evidence linking technology-related elements in hair with health outcomes remains sparse and methodologically heterogeneous. Maternal hair REEs were not associated with neural-tube-defect risk after adjustment for confounding at the relatively low concentrations examined [21]. Exploratory associations have been reported between hair REEs and nervous-system disease categories in children from the Kola Peninsula and between conventional hair metals and neuropsychological measures in Spanish children [20,60]. These studies differ in timing, outcome definition, exposure range and analytical panel and cannot establish a coherent dose–response relationship.

Health-outcome research will require prospective or clearly temporally ordered designs, prespecified hypotheses, sufficient power, control of multiple comparisons, assessment of nutritional and socioeconomic determinants, characterisation of co-exposures and independent replication. Most importantly, the biological matrix used as the exposure variable must first be shown to represent the relevant exposure process.

## 7. Multimatrix Evidence and Biomarker Validity

### 7.1. Analytical Feasibility Does Not Establish Biological Equivalence

The ability to measure an element in several biological specimens does not establish that those specimens represent the same exposure interval or biological process. Goullé et al. validated multielement ICP-MS procedures for whole blood, plasma, urine and hair, as well as reported population distributions for V, Ga, Ge, Rb, Sr, Pd, Ag, Sb, Ba, W, Pt, Bi, U and other elements [48]. This work is important because it demonstrates analytical feasibility across matrices, including elements rarely considered in routine biomonitoring. However, the blood, plasma and urine datasets included 100 measurements each, whereas the hair dataset included 45, and the study did not establish paired individual-level relationships between hair and internal fluids for the technology-related elements. The reported values are analytical and population comparators, not evidence that hair can substitute for blood or urine.

Olmedo et al. similarly demonstrated that several conventional metals can be measured reproducibly in blood, urine, saliva and hair [49]. Direct biological comparisons nevertheless reveal limited correspondence. Rodrigues et al. found only a weak hair–blood association for Pb and no meaningful relationships for Cu, Mn or Sr, while Semenova et al. showed that geographical patterns could differ between blood and hair [10,11]. In children, paired blood and urine measurements displayed only weak correspondence for As, Pb and Sr and essentially no relationship for Ba [33]. These findings do not invalidate any one matrix; rather, they demonstrate that matrix selection must be guided by toxicokinetics, exposure timing and the decision question.

Table 4 summarises the principal multimatrix studies informing the distinction between analytical feasibility, cross-matrix correspondence and validation of hair as a surrogate for internal exposure.

### 7.2. Element-Group Lessons from the Available Multimatrix Evidence

REEs and Th currently provide the clearest illustration of why group discrimination and quantitative dose estimation must be separated. Hair REEs repeatedly distinguish strongly contrasting mining and reference populations, but the most persuasive exposure–response evidence presently favours urine in occupationally exposed adults [14,15,16,17,18]. For Th, hair and urine both differentiated mine workers from local residents, yet only urinary measurements were linked to radiological dose through an established biokinetic model and no simple quantitative relationship between the two matrices was demonstrated [35]. The principal missing evidence is a paediatric study collecting hair, urine and blood from the same participants across a well-characterised REE or Th exposure gradient.

Sb and Ba show a second form of matrix specificity. Hair Sb was informative near an active mine but less informative in a geogenic exposure setting, while urinary speciation provided more direct evidence of internal processing [27,28,29]. For Ba, biomonitoring equivalents exist for urine and plasma, but paired paediatric blood and urine measurements showed no meaningful correspondence [31,33]. The implication is not merely that hair lacks a guidance value; it is that guidance values and biological interpretations cannot be transferred even between established internal matrices without evidence.

For PGEs and Ag, the evidence remains primarily descriptive. Hair can reveal geographical or demographic differences and may identify direct product contact, but no paediatric study currently links environmental concentration, hair concentration, an internal biomarker, absorbed dose and adverse effect [23,25,26]. Mn provides a useful cautionary analogue. Despite a much larger literature, a review of 86 studies found no universally reliable biological matrix for non-occupational environmental Mn exposure, and occupational studies indicate that group discrimination does not necessarily support individual classification [62,63]. Emerging technology-related elements should therefore not be expected to acquire individual-level interpretability solely through increased analytical sensitivity.

The overall evidence supports an element- and matrix-specific hierarchy. Hair is useful for exposure-pattern screening; blood or urine may provide stronger internal-exposure information for selected elements; and quantitative health interpretation requires a separate toxicokinetic and epidemiological evidence chain.

Table 5 integrates the most defensible interpretation of scalp-hair measurements for each element group with the complementary matrices currently supported by the evidence and the status of available matrix-specific biomonitoring assessment values [64,65,66,67,68]. Mercury (Hg) is included in the table solely as a comparison element and not as a target TTE. Methylmercury provides an instructive example in which hair interpretation is supported by a comparatively coherent evidence chain linking a defined chemical species and exposure pathway with blood–hair kinetics and health-risk interpretation [69,70]. Its inclusion therefore illustrates the level of element- and species-specific validation that is currently lacking for most TTEs; mercury-derived hair values are not transferable to these elements.

## 8. From Population Distributions to Health-Risk Interpretation

### 8.1. Reference Distributions and Health-Based Biomonitoring Values

A population reference distribution indicates how common or unusual a measurement is within a defined population, time period and analytical workflow. It does not indicate whether that measurement is safe, harmful or causally related to disease. Hair distributions are particularly sensitive to age, sex, geography, geology, hair pigmentation, nutritional status, cosmetic treatment, collection protocol, washing procedure, analytical platform, detection capability and statistical treatment.

Health-based biomonitoring values require a substantially stronger evidential chain. Frameworks developed through HBM4EU emphasise the need for a suitable biomarker, a toxicological or epidemiological point of departure, toxicokinetic linkage, explicit exposure assumptions, assessment factors, population applicability and confidence evaluation [64]. Santonen et al. similarly concluded that biomonitoring contributes to risk assessment only when the biomarker is fit for purpose, sampling and quality assurance are adequate and the relationship with exposure or effect is sufficiently characterised [65].

Biomonitoring equivalents illustrate this matrix specificity. Health Canada has used such values in screening-level assessments where adequate toxicokinetic information exists [66], and published equivalents for Ba relate specifically to urine and plasma [31]. The German Human Biomonitoring Commission likewise distinguishes statistical reference values from health-related HBM values [67]. Moreover, health-based interpretations may be revised when new evidence emerges, as demonstrated by the suspension of former blood-Pb HBM values when a defensible effect threshold could no longer be supported [68].

Hair Hg can contribute to methylmercury risk assessment because a comparatively coherent evidence chain connects a predominant chemical species, dietary exposure, blood and hair kinetics and epidemiological effects [69,70]. Clinical reference-interval principles also require carefully selected populations, sufficient sample size, appropriate partitioning and external verification [71]. Matrix-specific HBM guidance for Cd further demonstrates that values derived for blood or urine cannot be transferred to hair, even for the same element [72].

Within the literature and official biomonitoring frameworks reviewed, no health-based guidance value, biomonitoring equivalent, clinical action level, occupational biological limit or toxicity threshold validated specifically for paediatric scalp hair was identified for REEs, PGEs, Ag, Sb, Bi, Ba, Sr, V, U, Th, Rb, Ga, Ge, Nb, Ta or W. Matrix-specific assessment values were identified for selected elements in blood, plasma or urine, including biomonitoring equivalents for Ag, Ba and Bi and occupational biological reference values for Sb, Ba and V; however, these values were derived for other matrices, populations and interpretative purposes, and none can be transferred to paediatric scalp-hair interpretation without an element- and matrix-specific toxicokinetic derivation.

### 8.2. Why Hair Concentrations Cannot Be Converted into Hazard Quotients

A conventional non-cancer hazard quotient compares an estimated exposure dose with a health-based reference dose:

*HQ* = estimated exposure dose/health-based reference dose

Both quantities must represent compatible exposure metrics, commonly expressed as mass per kilogram of body weight per day. A hair concentration expressed as ng/g or µg/g does not directly represent daily intake, inhaled concentration, absorbed dose, circulating concentration, target-organ dose or cumulative body burden. Dividing a hair concentration by an oral reference dose therefore produces a numerical result without validated toxicological meaning.

Environmental guidelines are equally matrix-specific. Soil screening values, drinking-water limits and air guidelines apply to their corresponding media under defined assumptions concerning intake, frequency, duration and body weight. They cannot be compared numerically with hair concentrations. Environmental exposure modelling can legitimately accompany a hair study, but the resulting estimate should be described as a pathway-specific assessment, such as a soil-ingestion screening assessment, rather than as validation of a safe or unsafe hair value.

Direct hair-based quantitative risk assessment would require an element- and species-specific relationship between external exposure and hair, a validated relationship between hair and internal dose, a defined exposure window, quantitative control of external contamination, characterisation of population variability, relevant dose–response evidence and a validated hair guidance value. These conditions are not currently met for the technology-related elements considered here.

## 9. Weight-of-Evidence Interpretation and Public-Health Application

### 9.1. A Seven-Stage Interpretative Framework

The proposed framework is intended to prevent progression from analytical detection to a toxicological or clinical conclusion without the necessary intermediate evidence. The initial stage defines the decision question, distinguishing occurrence surveillance, population comparison, source investigation, intervention evaluation, selection for additional testing, formal risk assessment and clinical management. Hair cannot be assumed to simultaneously represent a historical exposure record, an environmental particle sampler, an internal-dose biomarker and a diagnostic test.

Analytical validity must then be established through verified sample identity, appropriate collection tools, standardised preparation, procedural blanks, interference control, calibration, precision, recovery, suitable LOD and LOQ, batch comparability and explicit censoring classification. Once analytical validity is secure, the population distribution can be described using detection and quantification frequencies, censoring-aware descriptive statistics, relevant age and sex strata, cosmetic-treatment information and review of extreme observations.

Source plausibility should subsequently be evaluated through geology, residence, school and other activity spaces, traffic, industrial activity, diet, drinking water, consumer products, medication, household occupations and measurements in soil, dust, air or water. Source attribution should remain probabilistic because a compatible hair pattern rarely proves a source independently. Multi-matrix corroboration can then be sought using blood, urine, nails, deciduous teeth, repeated hair, wash fractions or relevant environmental media, with the objective of achieving convergent evidence rather than identical numerical results.

Only after these stages should toxicological relevance be considered in relation to chemical species, particle characteristics, solubility, bioavailability, exposure route, critical effects, developmental susceptibility, human evidence and matrix-specific guidance values. Where the link with dose remains absent, interpretation should stop at exposure prioritisation or hazard identification. Proportionate action may range from no immediate intervention to analytical confirmation, recollection, environmental investigation, exposure reduction, validated clinical testing or specialist referral. Within Stage 6, “validated clinical testing” refers to confirmation using a clinically accepted, element- and species-appropriate biological test with an authoritative interpretative basis, typically in blood or urine where such testing exists, rather than by repeat hair analysis alone. “Specialist referral” refers to evaluation by an appropriate paediatric, clinical toxicology or environmental/occupational medicine service when confirmed exposure, compatible clinical findings or an authoritative matrix-specific action criterion warrants further clinical assessment. At every stage, insufficient evidence should trigger verification or additional data collection rather than automatic progression.

No single methodological feature or cross-matrix observation is sufficient by itself to validate scalp hair as a surrogate for internal dose; progression through the proposed framework therefore requires convergent evidence across analytical validity, source plausibility, complementary matrices and toxicological relevance.

**Figure 2:**
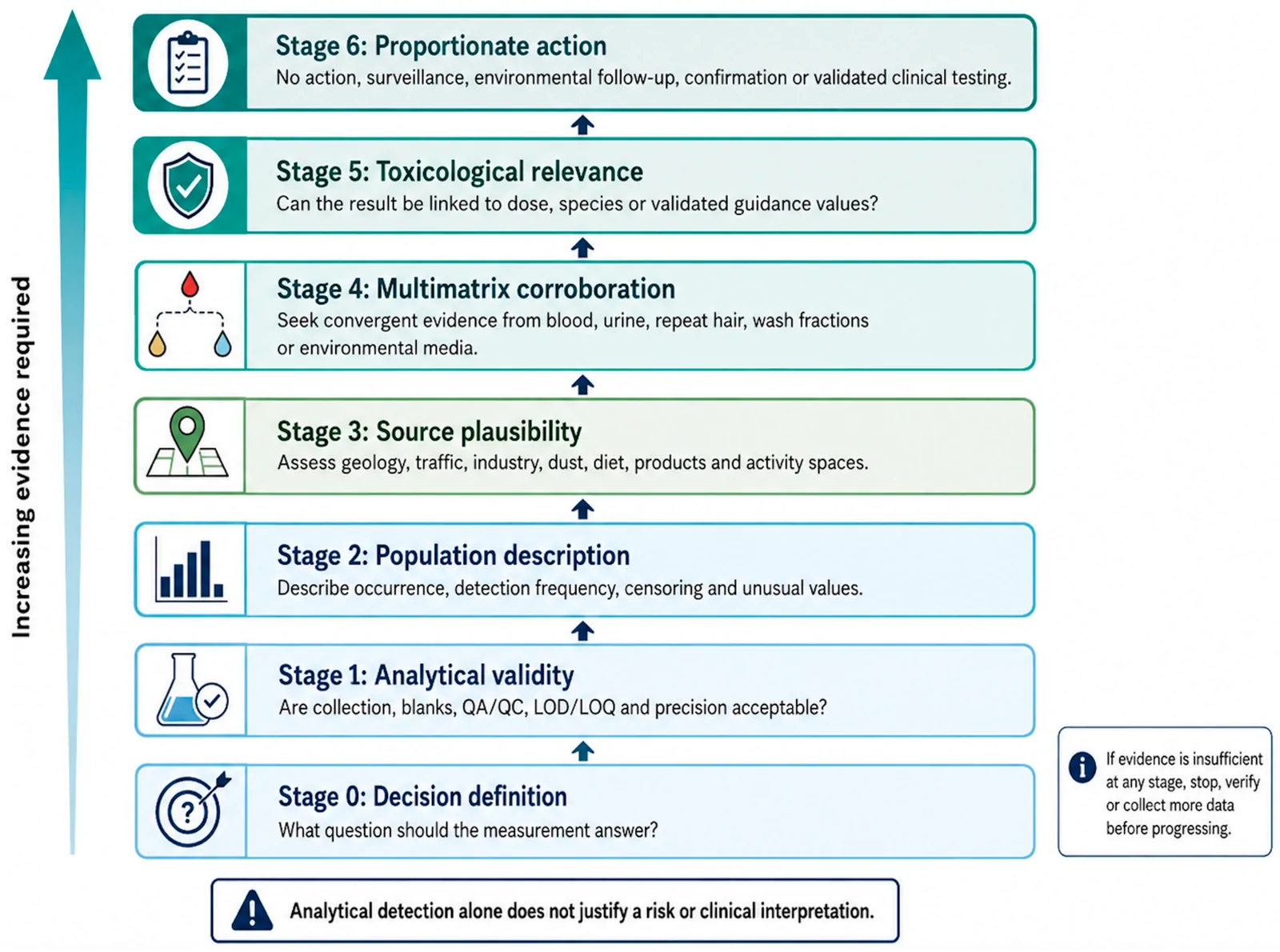
Seven-stage weight-of-evidence framework for interpreting technology-related trace elements in paediatric scalp hair. The framework progresses from definition of the decision question and analytical validity to population description, source plausibility, multi-matrix corroboration, toxicological relevance and proportionate action. Progression requires increasing evidence. Analytical detection alone does not justify a risk or clinical interpretation.

These seven stages, together with their core questions, evidential requirements and permissible conclusions or actions, are summarised in Table 6 and visualised in Figure 2.

### 9.2. Population Surveillance, Individual Results and Environmental Justice

The most defensible current public-health role of scalp hair for technology-related elements is structured screening. At population level, hair may identify geographical contrasts, demographic patterns, unusual upper-tail distributions, historical baselines, possible source profiles and groups requiring more detailed investigation. A coherent shift across a school or community may justify environmental follow-up even when no individual health threshold exists.

Individual interpretation requires greater caution. An isolated high result may reflect external deposition, cosmetic or product contact, unusual behaviour, sample misidentification, analytical interference, batch contamination or genuinely elevated exposure. Before communicating toxicological significance, the result should be confirmed analytically, participant metadata should be reviewed, a new sample should be considered and a validated internal matrix should be obtained where one exists. Terms such as “toxic level”, “poisoning”, “safe concentration” or “deficiency” should not be used without an authoritative matrix-specific basis.

Exposure assessment should also extend beyond residential address. Children divide their time among homes, schools, playgrounds, transport routes, sports facilities and other indoor and outdoor environments. Environmental measurements should correspond to those activity spaces and to the approximate period represented by the analysed hair segment.

These interpretative safeguards are particularly important in communities near mines, traffic corridors, industrial facilities or waste-management sites. Hair screening may reveal environmental inequalities, but overstating uncertain results can stigmatise communities, generate unnecessary anxiety and transfer responsibility for investigation to families. Community engagement, transparent explanation of uncertainty and access to appropriate environmental or clinical follow-up should therefore be integrated into study design.

## 10. Conclusions, Applications, and Prospects

Technology-related trace elements are increasingly relevant to environmental health as their use expands across renewable energy, digital infrastructure, transport, electronics, advanced materials and consumer products. Scalp hair offers important practical advantages for paediatric biomonitoring because sampling is non-invasive, readily repeated, compatible with multielement analysis and suitable for archived material. The evidence reviewed here nevertheless supports a more limited biological interpretation than these practical advantages might suggest. Hair can identify population distributions, geographical differences and source-related patterns, particularly where exposure differences are pronounced, but a measured concentration cannot generally be interpreted as a quantitative surrogate of absorbed dose or individual health risk.

The principal limitation is that the measured hair signal may combine follicular incorporation, deposition through sweat and sebum and external material retained on or within the shaft. The relative contribution of these processes is element-, species-, exposure- and hair-dependent and is further influenced by collection, washing, cosmetic treatment, analytical blanks, detection capability and statistical treatment. Multimatrix evidence reinforces this distinction: urine currently provides the clearest exposure–response evidence for long-term occupational REE exposure; hair and urine can both discriminate Th-exposed groups without demonstrating quantitative equivalence; Sb performance differs among exposure settings; and Ba has biomonitoring equivalents for urine and plasma but not for hair. Consequently, the most defensible present application of paediatric scalp-hair analysis is complementary screening and hypothesis generation within a weight-of-evidence assessment rather than stand-alone clinical or dose assessment.

The major research priority is direct paediatric validation linking well-characterised environmental sources with contemporaneous hair, urine and, where ethically and scientifically justified, blood measurements. Longitudinal and intervention studies should be prioritised over additional isolated cross-sectional surveys because within-person responses to remediation, relocation, traffic reduction, altered water supply or removal of a relevant product would provide stronger evidence of biomarker responsiveness. REE studies should evaluate individual elements and determine whether occupational urinary exposure–response relationships extend to lower paediatric exposures; PGEs and Ag require direct comparison with internal matrices; Sb requires greater emphasis on chemical speciation; Ba and Sr require paired-matrix studies; and repeated V measurements are needed to distinguish recent from longer-term exposure. Electronic-waste studies should integrate TTE mixtures with household or worksite dust, water, diet, parental occupation and child activity spaces.

Analytical development remains equally important. Future work should harmonise full procedural-blank control, contamination and interference assessment, LOD/LOQ definitions, reference materials, proficiency testing, washing protocols and censoring-aware statistical workflows. Hair-fibre morphology, treatment history and characteristics that may affect external retention or washing efficiency should be documented more consistently. Conventional total-element analysis should also be complemented, where toxicologically relevant, by particle-, species- or isotope-resolved approaches because chemical species, oxidation state, isotope composition, solubility, particle size and bioaccessibility may be more informative than total concentration alone. Species-resolved methods are particularly relevant to Sb, V and U, while radiological interpretation of U or Th requires appropriate isotope and biokinetic information rather than total hair concentration.

This review has several strengths, including integration of paediatric and occupational evidence, multimatrix comparisons, analytical-validity studies and biomonitoring frameworks, together with an explicit separation of analytical detection, exposure interpretation and toxicological interpretation. Its limitations should also be recognised. It is a structured critical narrative review rather than a formal systematic review or meta-analysis; the underlying literature is highly heterogeneous in population, source intensity, sampling period, hair preparation, analytical capability and statistical treatment, and no numerical risk-of-bias score was applied. Several target elements have been investigated in only one or a few paediatric populations, some of the strongest multimatrix evidence derives from occupationally exposed adults, and several publications from the author group derive from the same archived Spanish cohort and should therefore not be regarded as independent replications. Furthermore, much of the available literature measures total elemental content, and detailed hair morphology or ancestry-associated fibre characteristics have not been characterised consistently.

Population reference distributions may indicate whether a hair concentration is common or unusual within a specified population and analytical workflow, but they are not toxicity thresholds and should not be transferred between analytical methods or converted directly into dose or hazard quotients. Until direct paediatric multimatrix validation, stronger species-resolved evidence and matrix-specific health-based interpretation become available, scalp hair should not be presented as a stand-alone measure of absorbed dose, clinical toxicity or individual health risk. Used with explicit analytical safeguards, environmental context and proportionate multimatrix follow-up, however, it can provide a valuable and ethically attractive component of paediatric environmental surveillance.

## Figures and Tables

**Table 1:** Environmental relevance and defensible interpretative roles of scalp-hair measurements for selected technology-related trace elements.

Group	Examples	Selected Uses	Potential Sources	Defensible Role of Hair	Principal Limitation
Rare earth elements (REEs)	La–Lu, Y	Magnets, electronics, catalysts, phosphors, energy and medical technologies	Geogenic material; mining, beneficiation and smelting; industrial emissions; electronic waste	Spatial comparison, geochemical fingerprinting and source-pattern screening	External mineral particles, fractionation, low-level blanks and lack of hair–dose validation; urine currently has stronger exposure–response evidence in long-term occupational exposure
Platinum-group elements (PGEs)	Pt, Pd, Rh, Ru, Ir, Os	Vehicle and industrial catalysts, electronics, fuel cells and medical applications	Traffic particles, road dust, industrial emissions, mining and waste	Occurrence and area-comparison screening where analytical performance is demonstrated	Ultra-trace concentrations, blank contamination, instrumental carry-over/memory from residual analyte retained in the sample-introduction system, interferences and absence of validated hair thresholds
Silver	Ag	Electronics, photovoltaics, antimicrobial products, textiles and medical devices	Industrial and waste streams; products, jewellery, dyes, cosmetics and dust	Population occurrence and source/product-contact hypothesis generation	Direct contact and cosmetic treatment may dominate; no validated hair–dose or health threshold
Antimony and bismuth	Sb, Bi	Flame retardants, alloys, batteries, semiconductors, pharmaceuticals and cosmetics	Mining/smelting, indoor dust, brake wear, waste, medicines and consumer products	Context-specific screening with environmental and internal-matrix corroboration	Sb evidence varies by setting and discrete deposits may occur in hair; Bi is strongly confounded by products and medicines
Barium and strontium	Ba, Sr	Ceramics, glass, electronics, drilling fluids, pyrotechnics and specialised materials	Geology, soil, drinking water, diet, dust and selected industrial sources	Geogenic, dietary and spatial source-pattern assessment	Strong geogenic and dietary contributions; weak or absent cross-matrix correspondence; no hair-based guidance values
Vanadium	V	Steel alloys, catalysts and energy-storage technologies	Geology, residual-fuel combustion, industry, traffic mixtures and diet	Occurrence and source screening when detection frequency and exposure timing are adequate	Recent exposure, analytical censoring and source heterogeneity complicate interpretation; no validated hair guidance value
Uranium and thorium	U, Th	Nuclear fuel cycle and mineral-processing contexts	Geology, mining, phosphate materials, combustion residues and industry	Exposure-pattern screening and identification of highly exposed groups	Total concentration does not define isotope composition, speciation or radiological dose; dose estimation requires validated internal-matrix models
Sparsely studied technology-related elements	Rb, Ga, Ge, Nb, Ta, W	Semiconductors, fibre optics, capacitors, photovoltaics and advanced alloys	Geology, mining, manufacturing, combustion and electronic waste	Baseline occurrence, analytical method development and source-hypothesis generation	Human and paediatric evidence is sparse; analytical detectability currently exceeds biological interpretation

TTE, technology-related trace element. The categories are operational exposure groupings and do not imply shared toxicokinetics, toxicity or exclusively anthropogenic origin.

**Table 2:** Minimum reporting and validity checklist for paediatric scalp-hair studies of technology-related trace elements.

Domain	Minimum Requirement	Scientific Rationale
Decision question	Define whether the aim is occurrence, spatial comparison, source investigation, intervention follow-up, internal dose or clinical assessment.	Hair may be suitable for screening but not for every inferential objective.
Collection and sample definition	Report scalp region, proximal/distal orientation, segment length, mass, collection device and storage.	Collection devices and storage materials can contaminate ultra-trace panels; poorly defined segments weaken temporal interpretation.
Participant metadata	Record age, sex, hair colour, treatments, washing/swimming, products, medicines, diet, water, residence, school and activity spaces.	Biological, behavioural and product-related determinants can confound geographical comparisons.
Washing	Report reagents, sequence, duration, agitation, rinsing, drying and operational objective; analyse wash fractions where feasible.	No protocol completely separates endogenous from exogenous elements.
Digestion and instrumentation	Report vessels, acid grade, digestion programme, isotopes, reaction/collision conditions, calibration, internal standards, carry-over and washout.	Ultra-trace analyses are vulnerable to vessel contamination, interferences and instrumental carry-over/memory.
QA/QC	Use full procedural blanks, batch review, CRMs with assigned analytes, spikes, duplicates, calibration checks, isotope agreement and proficiency testing.	Validation for conventional certified elements does not validate unassigned technology-related elements.
LOD/LOQ and blank correction	Calculate from the full method in final hair units; retain negative blank-corrected values internally; distinguish ND, detected <LOQ, quantified and invalid results.	Instrument sensitivity alone is not a method detection capability; truncation biases distributions.
Censored data	Report detection frequencies and use censoring-aware methods; avoid arbitrary substitution and forced multivariate analysis.	Substitution can distort medians, correlations and apparent mixture or source patterns.
Biobanking	Document collection date, containers, storage and handling history, chain of custody and secondary-use ethics.	Archived hair is valuable, but historical comparability and contamination must remain auditable.
Environmental and temporal correspondence	Where source attribution is intended, characterise relevant soil, dust, air, water, food or product sources and document their spatial and temporal correspondence with the analysed hair segment.	Hair–environment associations are difficult to interpret when environmental samples represent different locations, activity spaces or time periods.
Interpretation and return of results	Separate population rarity, source plausibility, internal dose and health relevance; predefine confirmation, communication and referral pathways.	An unusual population value is not automatically toxic or clinically actionable.

CRM, certified reference material; LOD, limit of detection; LOQ, limit of quantification; ND, not detected; QA/QC, quality assurance and quality control.

**Table 3:** Selected reported concentrations of technology-related and comparator elements in paediatric scalp hair from European studies, with population and methodological characteristics affecting comparability.

Study and Setting	Population	Analytical Context	Selected Reported Concentrations	Detection/Censoring Information	Interpretative Note
Llorente Ballesteros et al., 2017 [58]; Madrid, Spain	*n* = 648; participants aged 0–18 years	ICP-MS; population reference study; sampling in 2008–2009	Medians: Ag 0.196 µg/g; Ba 0.500 µg/g; Bi 0.010 µg/g; Sr 1.29 µg/g	Element-specific detection frequencies were not reported in a directly harmonisable form for all selected endpoints.	Broad Madrid comparator spanning infancy to adolescence; age range and analytical workflow differ from the Alcalá cohorts.
Ruiz et al., 2023 [59]; Elche/Mediterranean Spain	*n* = 419; children aged 3–12 years	Washed hair; acid digestion; ICP-MS; reference-population pilot study	Medians: V 0.077 µg/g; Sr 3.14 µg/g; Ba 0.289 µg/g; Bi 0.005 µg/g; Ag and U 0.000 µg/g at the reported precision	The zero-valued Ag and U medians reflect reporting precision and the underlying detection structure and should not be interpreted as absence of exposure.	Useful independent Spanish comparator; conventional medians and very-low results are not equivalent to censoring-aware estimates.
Peña-Fernández et al., 2025 [25]; Alcalá de Henares, Spain	Children: *n* = 120, aged 6–9 years; adolescents: *n* = 97, aged 13–16 years	Archived 2001 scalp hair; Ag analysed by ICP-MS	Median Ag: children 0.1120 µg/g; adolescents 0.0695 µg/g	Ag detected in all children; 19.6% of adolescent results were < LOD.	Children showed a slightly higher median, whereas adolescents displayed a markedly wider upper tail; the age-group difference was not statistically significant. Cosmetic and product contact remain relevant.
Peña-Fernández et al., 2026 [30]; Alcalá de Henares, Spain	Children: *n* = 120, aged 6–9 years; adolescents: *n* = 97, aged 13–16 years	Archived 2001 scalp hair; Ba, Sr and V; censoring-aware treatment of V	Children: Ba 0.193 µg/g, Sr 0.412 µg/g and V 0.003 µg/g. Adolescents: Ba 0.287 µg/g, Sr 1.105 µg/g and V 0.011 µg/g (reported medians/censoring-aware estimates as applicable).	Children: Ba 38.3%, Sr 23.3% and V 74.2% < LOD. Adolescents: Ba 35.1%, Sr 37.1% and V 51.5% < LOD.	Demonstrates age-related concentration and quantifiability differences; values were interpreted as geogenic/urban tracers rather than toxicity thresholds.
Peña-Fernández et al., 2026 [36]; Alcalá de Henares, Spain—children	Up to *n* = 120, aged 6–9 years; element-specific analytical support varied with censoring	Archived 2001 scalp hair; REEs and technology-related elements by ICP-MS	Medians (µg/g): Ce 0.0109; La 0.0072; Nd 0.0042; Pr 0.00137; Gd 0.00068; Er 0.00035; Bi 0.006; Rb 0.0298; Sb 0.033; U 0.011	Y and Pt were not detected; Ir 99.2%, Pd 90.0%, Rh 95.0% and Th 95.8% < LOD.	One of the most extensive paediatric urban-baseline panels; highly censored endpoints support occurrence rather than continuous quantitative comparison.
Peña-Fernández et al., 2026 [36]; Alcalá de Henares, Spain—adolescents	Up to *n* = 97, aged 13–16 years; element-specific analytical support varied with censoring	Archived 2001 scalp hair; REEs and technology-related elements by ICP-MS	Selected medians (µg/g): Bi 0.002; Sb 0.0092; U 0.016	Most REEs and PGEs were extensively censored; element-specific detection frequencies and upper percentiles are required for interpretation.	Historical adolescent comparator; should not be collapsed with the child distribution or treated as a health-based interval.
Lo Medico et al., 2023 [23]; Sicily, Italy	108 adolescents aged 11–14 years were recruited; analytical *N* varied by element and site	Scalp hair; Pd and Pt reported at ng/g concentrations	Medians: industrial Pd 6.88 ng/g and Pt 0.44 ng/g; urban Pd 1.37 ng/g and Pt 1.56 ng/g; control Pd 0.56 ng/g and Pt 0.12 ng/g	Industrial Pd 72/Pt 70; urban Pd 22/Pt 22; control Pd 13/Pt 13.	Clear site contrasts support exposure-pattern screening; no paired internal matrix or health-based interpretation was established.

Values are reproduced using the statistics and units reported by each study. 1 ng/g = 0.001 µg/g. Arithmetic means, medians, percentiles and censoring-aware estimates are not interchangeable. This table is an evidence map, not a pooled European reference interval or a set of toxicity thresholds. Torrente et al. [60] and Esplugas et al. [61] provide important health-context and longitudinal evidence for conventional metals but are not repeated here because their panels do not provide directly comparable concentrations for the principal technology-related endpoints.

**Table 4:** Key multimatrix studies informing biomarker validity and cross-matrix interpretation.

Study	Population and Exposure Setting	Matrices	Cross-Matrix Result	What the Study Supports	What It Does Not Support
Skröder et al., 2017 [9]	Children; paediatric biomarker-validity analysis	Hair compared with established exposure indicators	Hair showed major limitations for several toxic and essential elements in children.	Hair performance must be demonstrated element by element in the target population.	Does not validate hair for emerging TTEs or as a universal internal-dose matrix.
Rodrigues et al., 2008 [10]	280 adults, Brazil	Hair, whole blood and plasma	Weak hair–blood relation for Pb; no meaningful relation for Cu, Mn or Sr.	Directly demonstrates limited correspondence between hair and circulating concentrations.	Adult population and conventional elements; findings cannot quantify TTE-specific hair kinetics.
Goullé et al., 2005 [48]	Reference/analytical populations; hair group *n* = 45	Whole blood, plasma, urine and hair	Validated multielement ICP-MS methods and reported matrix-specific distributions for several TTEs.	Establishes analytical feasibility across matrices.	Does not establish paired individual-level hair–blood or hair–urine equivalence or health-based hair values.
He et al., 2024 [18]	103 REE-exposed workers and 110 controls	Air, blood and urine	Urine showed stronger environmental correlations and exposure–response performance than blood.	Supports urine as a promising internal-exposure matrix for long-term occupational REE exposure.	Adult male occupational population; hair was not measured and paediatric transfer is untested.
Zhang et al., 2022 [35]	Rare-earth mine workers and nearby residents	Paired hair and urine for Th	Both matrices separated exposure groups, but no simple cross-matrix relationship was found; dose was estimated from urine.	Shows that group discrimination does not imply quantitative equivalence.	Adult/occupational setting; no paediatric health outcome or hair-based dose model.
Ye et al., 2018 [27]	Residents near active Sb mining	Environmental media, urine, saliva, hair and nails; speciation and micro-XRF	Hair related to estimated intake; urine/saliva characterised Sb species; discrete Sb-rich hair deposits were observed.	Supports multimatrix source and particle investigation in a high-exposure setting.	Endogenous incorporation and external particulate contributions were not fully separable.
Gebel et al., 1998 [28]	89 geogenically exposed and 47 reference participants	24 h urine, blood and scalp hair	No convincing elevation or soil–biomatrix relationship; urine was considered most practical.	Demonstrates that Sb biomarker performance is exposure-scenario dependent.	Older study and a different source regime from active mining.
Camacho-delaCruz et al., 2025 [33]	91 children aged 5–12 years, Mexico	Paired blood and urine	Weak correlations for As, Pb and Sr; essentially no correlation for Ba or most other elements.	Shows that even two internal matrices are not interchangeable and require element-specific validation.	Pilot sample; hair was not measured.

TTE, technology-related trace element. Analytical validation across matrices is distinct from validation of biological equivalence, internal-dose surrogacy or health-based interpretation.

**Table 5:** Biomarker-validity and matrix-specific assessment-value status of principal technology-related element groups.

Element/Group	Most Defensible Interpretation of Hair	Complementary Matrix/Evidence	Matrix-Specific Assessment-Value Status
REEs	Spatial/source-pattern screening; dose validity remains unestablished	Urine currently has the strongest exposure–response evidence in long-term occupational exposure; blood can discriminate exposure	No scalp-hair value identified; no qualifying target-group health-based biological value identified in the audited frameworks.
PGEs	Occurrence and area-comparison screening only	No universally validated matrix; blood or urine selection depends on compound, route and kinetics	No scalp-hair value identified for Pt, Pd, Rh, Ru, Ir or Os.
Ag	Occurrence and product/source-hypothesis generation	Blood or urine may be used for targeted questions, but validation is context dependent	No scalp-hair value identified. Whole-blood BE: 0.4 µg/L for ionic Ag; not transferable to hair and subject to greater paediatric uncertainty because the underlying PBPK model used adult physiology.
Sb	Context-dependent: positive mining findings but negative geogenic evidence	Urine, preferably with speciation, is generally more defensible for internal exposure	No health-based scalp-hair value identified. Urinary BAR: 0.2 µg/L; this is a statistical background/reference value, not a health-based limit.
Bi	Baseline occurrence and product/medicinal-context screening	Blood, plasma or urine selected according to medicinal or occupational context	No scalp-hair value identified. Published BEs: plasma 8.0 µg/L, whole blood 4.8 µg/L and urine 0.18 µg/L; derived from a therapeutic bismuth-subgallate intake scenario.
Ba	Geogenic, dietary and spatial patterning; not dose validated	Urine and plasma have published BEs; paired child blood–urine Ba showed no correlation	No scalp-hair value identified. Published BEs: urine 0.19 mg/L or 0.25 mg/g creatinine; plasma 9 µg/L. Urinary BAR: 10 µg/L for soluble Ba compounds.
Sr	Source-pattern interpretation; hair–blood equivalence unsupported	Paired child blood–urine data show only weak correlation; matrix choice requires validation	No scalp-hair value identified.
V	Occurrence/source screening where detection and timing are adequate	Urine is commonly used for recent occupational/environmental exposure, with timing limitations	No health-based scalp-hair value identified. Urinary BAR: 0.15 µg/L; this is a background/reference value, not a health threshold.
U and Th	Hair may separate highly exposed groups but does not define intake or dose	Urine is used for internal or radiological dose assessment in relevant exposure models	No scalp-hair value identified. Urinary U ranges may assist exposure interpretation but are not adverse-effect or clinical-action thresholds.
Rb, Ga, Ge, Nb, Ta and W	Occurrence, geochemical screening and method development	No preferred complementary matrix established for paediatric interpretation	No scalp-hair value or qualifying biological assessment value identified in the audited sources.
Hg, comparison element	Hair is interpretable for defined methylmercury pathways under validated assumptions	Hair and blood are established in specific methylmercury frameworks	Health-related hair values exist in defined methylmercury frameworks; they are not transferable to TTEs.

BAR, biological reference value; BE, biomonitoring equivalent; PBPK, physiologically based pharmacokinetic; TTE, technology-related trace element. Assessment values are reported only for the matrix and purpose for which they were derived. ‘No scalp-hair value identified’ refers to the literature and official frameworks audited up to 6 August 2026 and does not imply absence of hazard.

**Table 6:** Seven-stage weight-of-evidence framework for interpreting paediatric scalp-hair results.

Stage	Core Question	Required Evidence	Permissible Conclusion/Action
0. Decision definition	What decision should the measurement inform?	Explicit objective, target population, matrix role and exposure window	Redesign the study if hair cannot answer the decision question.
1. Analytical validity	Is the reported concentration technically credible?	Collection audit, blanks, interference control, precision, recovery, LOD/LOQ and batch review	Repeat, qualify or exclude analytically invalid results.
2. Population description	What was observed and how unusual is it?	Detection frequencies, censoring-aware summaries, relevant stratification and review of extreme observations	Describe occurrence and population rarity; do not infer toxicity.
3. Source plausibility	Which sources and pathways are compatible with the pattern?	Activity-space data, products, diet, geology, environmental matrices and elemental fingerprints	State probabilistic source hypotheses.
4. Multimatrix corroboration	Is there independent evidence of internal or environmental exposure?	Biologically and temporally appropriate blood, urine, nails, repeated hair, wash fractions or environmental matrices	Seek convergent evidence; discordance should redirect or narrow the exposure hypothesis rather than be treated automatically as analytical failure.
5. Toxicological relevance	Can the result be linked to dose or adverse effect?	Element/species-specific toxicokinetics, epidemiology, validated biomarkers and matrix-specific guidance values	Limit conclusions to hazard identification or exposure prioritisation when dose linkage is absent.
6. Proportionate action	What follow-up is justified by the total evidence?	Predefined confirmation, environmental investigation, communication and clinical-referral criteria	No action, surveillance, source control, confirmation using an appropriate validated clinical biomarker, or specialist referral when justified by confirmed exposure, clinical findings or authoritative matrix-specific criteria.

At any stage, insufficient evidence should trigger verification or additional data collection rather than automatic progression to a risk or clinical interpretation.

## Data Availability

No new primary data were created or analyzed in this study. The structured evidence matrices and biomonitoring-assessment-value audit generated as part of this review are provided in the Supplementary Materials. All underlying evidence and assessment values are available in the cited publications and official sources.

## Supplementary Materials

The following supporting information can be downloaded at https://www.mdpi.com/article/10.3390/toxics14090813/s1: Table S1A, structured evidence matrix of the core human and exposure-setting evidence underpinning biomarker validity and risk interpretation; Table S1B, structured evidence matrix of the core multimatrix, analytical and statistical-validity evidence; Table S1C, structured evidence matrix of the core biomonitoring and risk-interpretation frameworks; Table S2, formal element-by-element audit of health-based, screening and occupational biomonitoring assessment values for the technology-related trace elements considered; Table S3, source hierarchy, audit scope and classification rules. References [73,74,75,76,77,78,79,80,81], which support the assessment-value audit and source hierarchy presented in Tables S2 and S3, are cited in the Supplementary Materials and are included in the main reference list.

## Abbreviations

Ag, silver; As, arsenic; Ba, barium; BE, biomonitoring equivalent; Bi, bismuth; BLV, biological limit value; Cd, cadmium; CRM, certified reference material; Ga, gallium; Ge, germanium; HBM, human biomonitoring; HBM-GV, human-biomonitoring guidance value; Hg, mercury; HQ, hazard quotient; ICP-MS, inductively coupled plasma–mass spectrometry; ICP-MS/MS, inductively coupled plasma tandem mass spectrometry; Ir, iridium; LA-ICP-MS, laser-ablation inductively coupled plasma–mass spectrometry; LOD, limit of detection; LOQ, limit of quantification; Mn, manganese; Nb, niobium; Os, osmium; Pb, lead; Pd, palladium; PGE, platinum-group element; Pt, platinum; QA/QC, quality assurance and quality control; REE, rare earth element; Rh, rhodium; Ru, ruthenium; Sb, antimony; SEM-EDS, scanning electron microscopy with energy-dispersive X-ray spectroscopy; Sr, strontium; Ta, tantalum; Th, thorium; TTE, technology-related trace element; U, uranium; V, vanadium; W, tungsten; XRF, X-ray fluorescence; Y, yttrium.
