# Supplementary material for: Technology-Related Trace Elements in Paediatric Scalp-Hair Biomonitoring: Biomarker Validity, Environmental Relevance, and Implications for Risk Assessment
Source: Toxics. 2026 Sep 12;14(9):813. doi: 10.3390/toxics14090813 (PMC13611082; doi:10.3390/toxics14090813)
Supplement: Supplementary file 1 [file toxics-14-00813-s001.zip › toxics-4519013-supplementary.pdf]

Table S1A. Structured evidence matrix of the core human and exposure-setting evidence underpinning biomarker validity and risk interpretation.

Reference	Population/design	Exposure setting	Matrices/approach	Principal contribution	Principal limitation	Role in review
[14] Tong et al., 2004	Paediatric; 60 children aged 11–15 years	REE mining and reference areas, China	Scalp hair; comparison with mine/air patterns	Hair REEs were higher in the mining area and resembled environmental patterns	Strong spatial/source signal; no paired blood or urine and external particles were not excluded	Direct paediatric exposure-pattern evidence
[15] Wei et al., 2013	Human residents; mining area	REE mining region, China	Scalp hair	Higher REE concentrations and characteristic element patterns in the mining area	Limited internal-matrix validation; source and external-deposition contributions remain inseparable	Supporting mining-area hair evidence
[16] Meryem et al., 2016	Residents near mining/smelting	Hezhang, China	Agricultural soil, scalp hair and urine	Multimatrix REE contrasts and fractionation patterns	No validated individual-level hair–urine or dose relationship	Environmental-context and multimatrix evidence
[17] Dai et al., 2023	254 residents	REE smelting, mining and reference areas, China	Scalp hair with environmental context	Hair REEs differentiated exposure zones and displayed fractionation patterns	Spatial discrimination does not establish absorbed dose	Large human hair exposure-pattern study
[18] He et al., 2024	103 exposed workers and 110 controls	Long-term occupational REE exposure	Air, blood and urine	Urine showed stronger environmental correlations and exposure–response than blood	Adult male occupational population; hair not measured	Best current REE internal-matrix evidence
[19] Zielińska-Dawidziak et al., 2022	262 children/adolescents aged 5–19 years	Two regions of Madagascar	Scalp hair with limited environmental context	No clear regional contrast; weak associations with age and nutritional status	No internal matrix or health-based hair values	Paediatric background/context evidence
[20] Belisheva and Drogobuzhskaya, 2024	53 children aged 7–15 years	Vicinity of mining, Kola Peninsula	Scalp hair and clinical grouping	Exploratory associations between REEs and nervous-system disease categories	Small cross-sectional study; clinical heterogeneity and multiple comparisons	Hypothesis-generating health evidence
[21] Huo et al., 2017	191 affected pregnancies and 261 controls	North China	Maternal hair	Low maternal La, Ce, Pr and Nd were not associated with neural-tube-defect risk after adjustment	Maternal rather than paediatric matrix; low exposure range	Negative epidemiological evidence
[23] Lo Medico et al., 2023	Adolescents	Urban, petrochemical-industrial and comparison areas, Sicily	Scalp hair	Pd and Pt differed among areas	No paired internal matrix, inhaled-dose estimate or health endpoint	European PGE occurrence/source-screening evidence
[25] Peña-Fernández et al., 2025	120 children and 97 adolescents	Alcalá de Henares, Spain; archived 2001 samples	Scalp hair Ag with co-located topsoil context	Age/sex patterns and complete or near-complete detection depending on group	Historical single-city cohort; no paired blood/urine; soil and hair are non-equivalent matrices	Paediatric Ag occurrence and interpretative case study
[26] Galizia et al., 2024	Dyed and untreated human hair	Consumer/cosmetic exposure context	Scalp hair	Ag was higher in dyed hair	Not a paediatric exposure-gradient study; treatment alters the operational measurand	Direct evidence for cosmetic confounding

Reference	Population/design	Exposure setting	Matrices/approach	Principal contribution	Principal limitation	Role in review
[27] Ye et al., 2018	Residents near an active Sb mine	High-exposure mining area, China	Environmental media, urine, saliva, hair and nails; speciation and micro-XRF	Hair related to estimated intake; urine/saliva characterised methylated species; discrete Sb-rich deposits were observed	High-exposure setting; endogenous and particulate contributions not fully separable	High-value Sb multimatrix and particle evidence
[28] Gebel et al., 1998	89 geogenically exposed and 47 reference participants	Geogenic Sb exposure	24-h urine, blood and scalp hair	No convincing elevation or soil–biomatrix relationship; urine considered most practical	Older study and different source regime from active mining	Contrasting Sb biomarker evidence
[30] Peña-Fernández et al., 2026	Children and adolescents	Urban-industrial Spain; archived hair with topsoil V	Scalp hair Ba, Sr and V; co-located soil V	Demographic/spatial structure for Ba and Sr; V more extensively censored	Historical cohort; no paired internal matrices; V interpretation constrained by censoring	Paediatric geogenic/traffic-tracer case study
[31] Poddalgoda et al., 2017	General-population risk-assessment derivation	Barium	Urine and plasma toxicokinetic modelling	Derived Ba biomonitoring equivalents for urine and plasma	Values are matrix-specific and not hair or paediatric clinical thresholds	Positive example of matrix-specific BE derivation
[32] Zeng et al., 2022	3707 participants aged 8–17 years	NHANES 2003–2018	Urinary Ba and blood-pressure measures	Cross-sectional associations with selected blood-pressure measures	Cross-sectional design; does not validate hair or establish causality	Paediatric internal-matrix epidemiology
[33] Camacho-delaCruz et al., 2025	91 children aged 5–12 years	School-aged biomonitoring, Mexico	Paired blood and urine	Weak correlations for As, Pb and Sr; essentially no correlation for Ba	Pilot sample; hair not measured	Direct evidence that internal matrices are not interchangeable
[34] Pérez et al., 2018	120 children aged 6–11 years	Valencian Region, Spain	First-morning urine and dietary data	Dietary groups were associated with several urinary trace elements	Short-term dietary influence and spot-urine limitations	Evidence for diet as a core covariate
[35] Zhang et al., 2022	Rare-earth mine workers and nearby residents	REE mining area	Paired hair and urine for Th	Both matrices separated exposure groups; no simple cross-matrix relation; dose estimated from urine	Adult/occupational context; no paediatric health outcome	Most informative paired hair–urine TTE study
[36] Peña-Fernández et al., 2026	120 children and 97 adolescents	Alcalá de Henares, Spain; archived 2001 samples	Scalp hair REEs, PGEs and other TTEs	Historical urban occurrence, age-related patterns and censoring-aware interpretation	Single historical cohort; no internal-matrix validation	Broad paediatric TTE baseline
[37] Akram et al., 2025	Informal e-waste workers and comparison groups	Pakistan	Scalp hair	Workers showed higher selected conventional metals	Adult occupational study; emerging TTEs and paired internal matrices limited	Electronic-waste screening evidence
[51] Drobyshev et al., 2017	166 schoolchildren	Near a toxic-waste site and comparison areas	Scalp hair	Multielement spatial patterns	No paired internal matrix; modest area groups and external contamination uncertainty	School-based spatial screening evidence

Reference	Population/design	Exposure setting	Matrices/approach	Principal contribution	Principal limitation	Role in review
[52] Batyrova et al., 2025	1595 schoolchildren	Oil-producing region, Kazakhstan	Scalp hair	Large-cohort age, sex and geographical patterns	Distance to nominal sources did not consistently predict concentrations; no paired environmental/internal matrices	Large paediatric pattern study
[53] Beltrán-Ardila et al., 2026	69 pooled samples from 14 schools	Bogotá, Colombia	Pooled scalp hair	School-level multielement spatial heterogeneity	Pooling prevents individual distributions and individual risk inference	Low-cost surveillance model
[54] Peña-Fernández et al., 2014	Spanish children aged 6–9 years	Alcalá de Henares, Spain	Scalp hair and urban topsoil	Descriptive population values and source hypotheses	The term reference value is statistical, not health-based; soil–hair equivalence not established	Foundational paediatric hair/reference study
[55] Peña-Fernández et al., 2014	Children and adolescents	Alcalá de Henares, Spain	Scalp hair Pb	Age, sex and residential-area patterns	Hair Pb is not a clinical blood-Pb surrogate	Developmental and spatial case study
[56] Peña-Fernández et al., 2016	Children and adolescents	Alcalá de Henares, Spain	Scalp hair and urban topsoil	Age and residential effects across multiple elements	Cross-sectional, one-city design; no paired internal matrices	Population determinants and source-context evidence
[57] Peña-Fernández et al., 2017	Healthy adolescents aged 13–16 years	Alcalá de Henares, Spain	Scalp hair	Sex-related variability across metals and metalloids	Single population and multiple comparisons	Evidence for demographic partitioning
[58] Llorente Ballesteros et al., 2017	648 participants aged 0–18 years	Madrid, Spain	Scalp hair; ICP-MS	Large European paediatric reference distributions	Wide age span and method-specific distributions; not health thresholds	European comparison dataset
[59] Ruiz et al., 2023	419 children aged 3–12 years	Mediterranean Spain	Washed scalp hair; ICP-MS	Reference distributions for 28 elements	Pilot regional population; very-low results and detection structure must be retained	Independent European comparison dataset
[60] Torrente et al., 2014	Children	Spain	Scalp hair and neuropsychological assessment	Exploratory metal–behaviour associations	Preliminary cross-sectional associations and biomarker uncertainty	Health-outcome hypothesis generation
[61] Esplugas et al., 2019	Schoolchildren; repeated community surveys	Near a hazardous-waste incinerator, Catalonia	Scalp hair over approximately 20 years	Long-term temporal biomonitoring patterns	Changing cohorts and exposure context; hair-specific causal interpretation limited	Rare longitudinal community evidence

This table maps the core human evidence used to evaluate exposure patterning, source plausibility, paediatric applicability and health interpretation; it is not an exhaustive catalogue of all contextual literature cited in the review.

Table S1B. Structured evidence matrix of the core multimatrix, analytical and statistical-validity evidence.

Reference	Design	Matrices/topic	Principal contribution	Interpretative implication	Principal limitation	Role in review
[4] Martinez-Morata et al., 2023	State-of-the-science review	Multiple metals and biological matrices	Element- and matrix-specific biomarker performance	No universal matrix; toxicokinetics and exposure window determine fitness	Broad review rather than TTE-specific paediatric validation	Core biomarker-selection framework
[5] Esteban and Castaño, 2009	Critical review	Non-invasive matrices	Advantages and limitations of hair and other specimens	Supports complementary rather than universal use of hair	Predates much current TTE analytical evidence	Foundational non-invasive matrix review
[6] Pozebon et al., 2017	Analytical review	Elemental hair analysis	Sampling, washing, digestion, instrumentation and interpretation	Documents major sources of interstudy variability	Broad element scope and variable underlying evidence	Core hair-analysis methodology review
[7] Kempson and Lombi, 2011	Critical review	Hair biology, toxicology and health applications	Mechanistic routes and interpretative limitations	Clarifies multiple pathways into and onto hair	Not focused on paediatric TTEs	Mechanistic hair framework
[8] Bencko, 1995	Review	Occupational/environmental hair biomonitoring	Hair may support screening and geographical delineation	Warns against universal biomarker interpretation	Older analytical context	Historical conceptual foundation
[9] Skröder et al., 2017	Paediatric multimatrix study/critical analysis	Hair versus established exposure indicators	Major limitations of hair for several toxic and essential elements in children	Directly challenges unvalidated paediatric hair interpretation	Conventional rather than emerging TTE panel	High-value paediatric validity evidence
[10] Rodrigues et al., 2008	280 adults	Hair, whole blood and plasma	Weak hair–blood Pb relation; no relation for Cu, Mn or Sr	Direct evidence against assuming circulating equivalence	Adult population and conventional elements	Direct cross-matrix validation evidence
[11] Semenova et al., 2019	Populations in polluted regions	Hair and blood	Geographical contrasts differed between matrices	Matrix choice changes the observed exposure pattern	Cross-sectional and population-specific	Cross-matrix pattern evidence
[38] Petrick et al., 2020	Methods review	Minimally invasive paediatric biospecimens	Feasibility of combining hair with other child-friendly matrices	Supports multimatrix exposome designs	Not specific to TTE validity	Paediatric collection framework
[41] Lermen et al., 2020	Biobanking comparison	HBM and clinical biorepositories	Need for harmonised storage, shipment and metadata	Supports quality requirements for archived hair	Not element-specific	Biobanking/archival framework
[42] Runkel et al., 2026	Analytical method optimisation	Human hair; triple-quadrupole ICP-MS	Collection tools and vessel materials can affect ultratrace results	Demonstrates that method contamination can dominate emerging-element signals	Method-focused; biomarker validity not assessed	High-priority ultratrace QA/QC evidence
[43] Morton et al., 2002	Experimental washing comparison	Human hair; ICP-MS	Washing procedures removed exogenous elements with different efficiencies	No universal removal of external material	Older instrument/method context	Core washing evidence
[44] Verrey et al., 2019	Method-development study	Human hair washing for inorganic elements	Proposed a sequential washing protocol	Improves reproducibility but still defines an operational fraction	Does not prove purely endogenous measurement	Contemporary washing evidence

Reference	Design	Matrices/topic	Principal contribution	Interpretative implication	Principal limitation	Role in review
[45] David et al., 2023	LA-ICP-MS method study	Spatial hair analysis	Pre-cleaning was not beneficial for every chronic-exposure application	Analytical objective determines whether washing is appropriate	Not directly comparable with bulk digestion	Spatial-analysis counterpoint
[47] Tadić et al., 2025	QA/QC review	Human biomonitoring reference materials	Identified gaps in matrix-matched reference materials and proficiency testing	Especially relevant to emerging elements lacking assigned values	Broad HBM scope	Reference-material framework
[48] Goullé et al., 2005	Analytical validation/reference study	Whole blood, plasma, urine and hair	Validated multielement ICP-MS and reported matrix-specific distributions	Measurement feasibility does not establish paired biological equivalence	Hair group n=45; matrices not used to validate individual cross-matrix relations	Key analytical-versus-biological distinction
[49] Olmedo et al., 2010	Analytical validation	Blood, urine, saliva and hair	Validated multi-matrix methods for selected conventional metals	Analytical comparability is necessary but not sufficient for biomarker equivalence	Not focused on emerging TTEs	Supporting multimatrix method evidence
[50] Helsel, 2012	Statistical monograph	Left-censored environmental data	Documents bias from arbitrary substitution and appropriate censored-data methods	Methods require adequate sample size and assumptions	Methodological rather than element-specific	Core censoring framework
[62] Shilnikova et al., 2022	Critical review of 86 studies	Environmental Mn biomarkers	No universally reliable matrix for non-occupational Mn exposure	Even a well-studied element lacks simple universal biomarker validity	Mn is a cautionary analogue, not a target TTE	Analogue for emerging-element caution
[63] Bader et al., 1999	Occupational study	Blood, urine and axillary hair for Mn	Some group-level discrimination but weak individual interpretability	Group contrast does not validate individual classification	Occupational Mn analogue	Direct analogue for group-versus-individual inference

Analytical feasibility and method validation are separated from biological equivalence and health-based interpretability.

Table S1C. Structured evidence matrix of the core biomonitoring and risk-interpretation frameworks.

Reference	Framework/design	Scope	Principal contribution	Implication for hair interpretation	Principal limitation	Role in review
[64] Apel et al., 2020	HBM4EU methodological framework	Derivation of HBM guidance values	Defines biomarker, point-of-departure, toxicokinetic and uncertainty requirements	Health-based values are matrix- and population-specific	General framework; not hair/TTE-specific	Primary HBM-GV derivation framework
[65] Santonen et al., 2023	HBM4EU risk-assessment guidance	Use of HBM in chemical risk assessment	Biomarkers must be fit for purpose and supported by QA/QC and dose-response evidence	Prevents use of unvalidated hair values in risk calculations	General framework	Core risk-interpretation framework
[66] Poddalgoda et al., 2024	Health Canada review	Biomonitoring-based risk assessment	Describes BE use as screening/prioritisation rather than diagnosis	Reinforces matrix specificity and uncertainty	National programme context	Regulatory biomonitoring context
[67] Schulz et al., 2011	German HBM Commission update	Reference and HBM values	Distinguishes statistical reference values from toxicologically derived HBM values	Reference percentiles have no intrinsic health meaning	Values evolve with evidence	Essential terminology framework
[68] Wilhelm et al., 2010	German HBM Commission reassessment	Blood lead	Suspension of previous HBM values when a defensible effect threshold was no longer supported	Shows health-based values are revisable and evidence-dependent	Lead example rather than target TTE	Cautionary regulatory example
[69] Domínguez-Morueco et al., 2022	European HBM risk assessment	Hair mercury	Hair Hg can support methylmercury risk assessment under defined assumptions	Valid element-specific exception, not a precedent for all metals	Species/pathway-specific	Positive hair-validity comparator
[70] McDowell et al., 2004	NHANES population study	Hair mercury in children and women	Provides population reference data for a validated pathway	Reference data remain distinct from a universal toxicity threshold	Hg-specific	Population comparator for a validated hair endpoint
[71] CLSI EP28-A3c	Clinical laboratory guideline	Reference intervals	Defines reference-population, partitioning and verification principles	Supports careful descriptive intervals but not toxicological reinterpretation	Clinical analyte context differs from environmental hair	Reference-interval methodology
[72] Lamkarkach et al., 2021	HBM4EU value derivation	Cadmium in blood and urine	Demonstrates matrix-specific derivation of health-based guidance values	Cannot be transferred to hair even for the same element	Cadmium comparator rather than target TTE	Positive matrix-specific HBM example

Framework and comparator studies define the evidential requirements for matrix-specific biomonitoring values and risk interpretation.

Table S2. Formal element-by-element audit of health-based, screening and occupational biomonitoring assessment values for the technology-related trace elements considered.

Element/group	Health-based or screening biological assessment value identified	Occupational biological assessment/reference value	Validated paediatric scalp-hair value	Implication for the review	Key audited sources
REEs (La–Lu and Y)	No qualifying health-based value identified for blood, urine or hair in the audited frameworks. DFG lists Gd with BAR not established.	No BAT/BLW/BMGV identified for the group; Gd BAR not established.	None identified.	Hair supports spatial/source-pattern screening only. Group and individual REEs require matrix-specific validation.	UBA HBM; DFG 2025; HBM4EU/PARC framework
Pt	No health-based biological value identified.	No occupational biological assessment value identified. Air limits for chloroplatinates were excluded.	None identified.	Hair Pt is an occurrence/source-screening endpoint, not a dose or risk metric.	DFG 2025; HSE BMGV; UBA HBM
Pd	No health-based biological value identified.	No occupational biological assessment value identified.	None identified.	No validated hair–internal-dose relationship.	DFG 2025; HSE BMGV; UBA HBM
Rh	No health-based biological value identified.	No occupational biological assessment value identified.	None identified.	No validated paediatric hair interpretation.	DFG 2025; HSE BMGV; UBA HBM
Ru	No health-based biological value identified.	No occupational biological assessment value identified.	None identified.	No validated paediatric hair interpretation.	DFG 2025; HSE BMGV; UBA HBM
Ir	No health-based biological value identified.	No occupational biological assessment value identified.	None identified.	No validated paediatric hair interpretation.	DFG 2025; HSE BMGV; UBA HBM
Os	No health-based biological value identified.	No occupational biological assessment value identified.	None identified.	No validated paediatric hair interpretation.	DFG 2025; HSE BMGV; UBA HBM
Ag	Whole-blood BE: 0.4 µg/L for ionic Ag, associated with the US EPA RfD and protection against argyria. Health Canada notes greater uncertainty for children because the PBPK model used adult physiology.	No BAT/BLW/BMGV or BAR identified in the audited occupational lists.	None identified.	The whole-blood BE is not transferable to hair. Hair Ag is additionally vulnerable to product and cosmetic contact.	Health Canada 2016/2022; DFG 2025; HSE BMGV
Sb	No health-based HBM value or BE identified in the audited sources.	DFG urine BAR: 0.2 µg/L. EKA not established. HSE states that no published occupational biological monitoring guidance value is available; background levels can distinguish exposure.	None identified.	BAR is a statistical background value, not a health limit. Urine/speciation is more defensible for internal exposure than hair.	DFG 2025; HSE 2025; UBA HBM

Element/group	Health-based or screening biological assessment value identified	Occupational biological assessment/reference value	Validated paediatric scalp-hair value	Implication for the review	Key audited sources
Bi	Published matrix-specific BEs associated with the bismuth-subgallate therapeutic intake used in the derivation study: plasma 8.0 μg/L; whole blood 4.8 μg/L; urine 0.18 μg/L. The derivation authors noted that no general-population exposure guidance value was available.	No BAT/BLW/BMGV or BAR identified in the audited occupational lists.	None identified.	These values are screening estimates for adult plasma, whole blood and urine based on a medicinal-intake scenario; they are neither paediatric scalp-hair values nor clinical action levels.	Poddalgoda et al., 2020; DFG 2025; HSE BMGV
Ba	Published BEs: urine 0.19 mg/L or 0.25 mg/g creatinine and plasma 9 μg/L. Health Canada uses a urinary guidance value of 246 μg/g creatinine in its biomonitoring-based approach.	DFG urine BAR: 10 μg/L for soluble Ba compounds.	None identified.	BE and BAR values are matrix- and purpose-specific and cannot be applied to hair.	Poddalgoda et al., 2017; Health Canada; DFG 2025
Sr	No qualifying health-based biological value identified.	No BAT/BLW/BMGV or BAR identified in the audited occupational lists.	None identified.	Weak blood–urine and absent hair–blood correspondence argue against matrix transfer.	DFG 2025; HSE BMGV; UBA HBM
V	No health-based HBM value or BE identified in the audited sources.	DFG urine BAR: 0.15 μg/L. BAT and EKA not established.	None identified.	BAR is a background/reference value, not a health threshold. Hair V requires detection- and timing-aware interpretation.	DFG 2025; UBA HBM; Health Canada science-approach inventory
U	ATSDR identifies urine as the preferred biological matrix for assessing uranium exposure. High urinary uranium concentrations may indicate substantial recent exposure; the ATSDR toxicological profile reports occupational data in which concentrations >100 μg/L were considered indicative of recent absorption, whereas none of 600 non-uranium-worker controls exceeded 40 μg/L. These concentrations are exposure-context data and not health-based action thresholds.	DFG BAR not established for hardly soluble or soluble inorganic uranium compounds.	None identified.	Urinary uranium can support assessment of internal exposure, whereas total uranium in scalp hair does not provide a validated measure of absorbed dose or radiological risk. Reported urinary concentrations must not be interpreted as adverse-effect thresholds.	ATSDR/CDC; DFG 2025
Th	No qualifying health-based biological value identified.	No occupational biological assessment value identified in the audited sources.	None identified.	Urinary modelling, not hair, supported dose estimation in the available paired study.	DFG 2025; HSE BMGV; UBA HBM
Rb	No qualifying health-based biological value identified.	No occupational biological assessment value identified.	None identified.	Hair Rb should remain an occurrence/geochemical endpoint.	DFG 2025; HSE BMGV; UBA HBM

Element/group	Health-based or screening biological assessment value identified	Occupational biological assessment/reference value	Validated paediatric scalp-hair value	Implication for the review	Key audited sources
Ga	No qualifying health-based biological value identified.	No occupational biological assessment value identified for elemental Ga in the audited sources.	None identified.	Analytical occurrence currently exceeds biological interpretation.	DFG 2025; HSE BMGV; UBA HBM
Ge	No qualifying health-based biological value identified.	No occupational biological assessment value identified.	None identified.	Analytical occurrence currently exceeds biological interpretation.	DFG 2025; HSE BMGV; UBA HBM
Nb	No qualifying health-based biological value identified.	No occupational biological assessment value identified.	None identified.	Analytical occurrence currently exceeds biological interpretation.	DFG 2025; HSE BMGV; UBA HBM
Ta	No qualifying health-based biological value identified.	No occupational biological assessment value identified.	None identified.	Analytical occurrence currently exceeds biological interpretation.	DFG 2025; HSE BMGV; UBA HBM
W	No qualifying health-based biological value identified.	No occupational biological assessment value identified in the audited sources.	None identified.	Analytical occurrence currently exceeds biological interpretation; hair W should remain an occurrence and method-development endpoint.	DFG 2025; HSE BMGV; UBA HBM

Values are reported for the biological matrix and interpretative purpose for which they were derived. BE values are screening values linked to an external health-protective exposure value and are not clinical diagnostic thresholds. BAR values are statistical occupational background/reference values and are not health-based limits. 'None identified' refers to the source hierarchy in Table S3, searched up to 6 August 2026.

Table S3. Source hierarchy, audit scope and classification rules.

Priority/source class	Sources searched	Purpose	Audit treatment/outcome
1. Official HBM authorities	German Environment Agency HBM Commission; HBM4EU/PARC methodological outputs	Current health-based HBM values, derivation principles and statistical reference-value concepts	No target-element paediatric scalp-hair value identified. Statistical reference values were not treated as health thresholds.
2. National biomonitoring risk assessments	Health Canada / Environment and Climate Change Canada official assessments	Biomonitoring equivalents and population-level screening assessments	Ag whole-blood BE and Ba and Bi matrix-specific BEs were identified; all are non-hair, purpose-specific assessment values.
3. Occupational biological assessment systems	DFG List of MAK and BAT Values 2025	BAT, BLW, BAR and EKA entries	Urinary BARs were identified for Sb, Ba and V; Gd and U BARs were not established. BARs were classified as background/reference values, not health-based limits.
4. United Kingdom occupational guidance	Health and Safety Executive biological-monitoring guidance and Sb-specific monitoring information	Published BMGVs and practical biological-monitoring guidance	No target-element BMGV was identified; HSE reports no published occupational biological-monitoring guidance value for Sb.
5. Clinical and environmental toxicology authorities	ATSDR uranium Public Health Statement and Toxicological Profile	Exposure biomarkers and toxicological/clinical interpretation	Urinary U was retained as an exposure-assessment matrix; exposure-context concentrations were not classified as health-based action values.
6. Peer-reviewed BE derivations	Primary Ba and Bi biomonitoring-equivalent derivations in Regulatory Toxicology and Pharmacology	Matrix-specific derivation assumptions and numerical values	Ba and Bi BEs were retained with their original matrices, populations and purposes; no transfer to hair was permitted.
7. Excluded value types	Commercial laboratory ranges; descriptive study percentiles; hair 'normal ranges'; environmental-medium standards; air-only occupational limits	Non-authoritative, non-biological or matrix-incompatible comparators	Excluded from health-based columns. They may be described only as population, environmental or occupational context.

The audit is an evidence-classification exercise, not a declaration that unlisted values can never exist. All conclusions are limited to the named sources and the audit date.

References cited in Supplementary Tables

[73] German Environment Agency. Reference and HBM Values. Updated 30 July 2026. Available online: https://www.umweltbundesamt.de/en/topics/health/commissions-working-groups/human-biomonitoring-commission/reference-hbm-values (accessed on 6 August 2026).

[74] German Research Foundation—Permanent Senate Commission for the Investigation of Health Hazards of Chemical Compounds in the Work Area. List of MAK and BAT Values 2025. Maximum Concentrations at the Workplace and Assessment Values in Biological Material. Report 61; German Research Foundation: Bonn, Germany, 2025. https://doi.org/10.34865/mbwl_2025_eng.

[75] Health and Safety Executive. Biological Monitoring Guidance Values. Available online: https://books.hse.gov.uk/biological-monitoring-guidance-values (accessed on 6 August 2026).

[76] Health and Safety Executive. Antimony (Urine), 2025. Available online: https://books.hse.gov.uk/product/9780717667642 (accessed on 6 August 2026).

[77] Health Canada. Science Approach Document: Biomonitoring-Based Approach 2 for Barium-Containing Substances, Molybdenum-Containing Substances, Silver-Containing Substances, Thallium-Containing Substances and Inorganic Tin-Containing Substances; Government of Canada: Ottawa, ON, Canada, 2016. Available online: https://www.canada.ca/en/environment-climate-change/services/evaluating-existing-substances/science-approach-document-biomonitoring-based-approach-2barium-containing-substances-molybdenum-containing-s.html (accessed on 6 August 2026).

[78] Environment and Climate Change Canada; Health Canada. Screening Assessment: Silver and Its Compounds; Government of Canada: Ottawa, ON, Canada, 2022; Cat. No. En84-302/2022E-PDF; ISBN 978-0-660-44697-4. Available online: https://www.canada.ca/en/environment-climate-change/services/evaluating-existing-substances/screening-assessment-silver-compounds.html (accessed on 6 August 2026).

[79] Poddalgoda, D.; Hays, S.M.; Nong, A. Derivation of Biomonitoring Equivalents (BE Values) for Bismuth. Regul. Toxicol. Pharmacol. 2020, 114, 104672. https://doi.org/10.1016/j.yrtph.2020.104672.

[80] Agency for Toxic Substances and Disease Registry. Public Health Statement for Uranium; U.S. Department of Health and Human Services, Public Health Service: Atlanta, GA, USA, 2013. Available online: https://www.atsdr.cdc.gov/toxprofiles/tp150-c1-b.Pdf (accessed on 6 August 2026).

[81] Agency for Toxic Substances and Disease Registry. Toxicological Profile for Uranium; U.S. Department of Health and Human Services, Public Health Service: Atlanta, GA, USA, 2013. Available online: https://www.atsdr.cdc.gov/toxprofiles/tp150.pdf (accessed on 6 August 2026).
